# Measurement of masses in the $\mathrm{t}\overline{\mathrm{t}}$ system by kinematic endpoints in pp collisions at $\sqrt{s}=7\ \mathrm{TeV}$

**DOI:** 10.1140/epjc/s10052-013-2494-7

**Published:** 2013-07-16

**Authors:** The CMS Collaboration, S. Chatrchyan, V. Khachatryan, A. M. Sirunyan, A. Tumasyan, W. Adam, T. Bergauer, M. Dragicevic, J. Erö, C. Fabjan, M. Friedl, R. Frühwirth, V. M. Ghete, N. Hörmann, J. Hrubec, M. Jeitler, W. Kiesenhofer, V. Knünz, M. Krammer, I. Krätschmer, D. Liko, I. Mikulec, D. Rabady, B. Rahbaran, C. Rohringer, H. Rohringer, R. Schöfbeck, J. Strauss, A. Taurok, W. Treberer-Treberspurg, W. Waltenberger, C.-E. Wulz, V. Mossolov, N. Shumeiko, J. Suarez Gonzalez, S. Alderweireldt, M. Bansal, S. Bansal, T. Cornelis, E. A. De Wolf, X. Janssen, A. Knutsson, S. Luyckx, L. Mucibello, S. Ochesanu, B. Roland, R. Rougny, H. Van Haevermaet, P. Van Mechelen, N. Van Remortel, A. Van Spilbeeck, F. Blekman, S. Blyweert, J. D’Hondt, A. Kalogeropoulos, J. Keaveney, M. Maes, A. Olbrechts, S. Tavernier, W. Van Doninck, P. Van Mulders, G. P. Van Onsem, I. Villella, B. Clerbaux, G. De Lentdecker, A. P. R. Gay, T. Hreus, A. Léonard, P. E. Marage, A. Mohammadi, T. Reis, L. Thomas, C. Vander Velde, P. Vanlaer, J. Wang, V. Adler, K. Beernaert, L. Benucci, A. Cimmino, S. Costantini, S. Dildick, G. Garcia, B. Klein, J. Lellouch, A. Marinov, J. Mccartin, A. A. Ocampo Rios, D. Ryckbosch, M. Sigamani, N. Strobbe, F. Thyssen, M. Tytgat, S. Walsh, E. Yazgan, N. Zaganidis, S. Basegmez, G. Bruno, R. Castello, A. Caudron, L. Ceard, C. Delaere, T. du Pree, D. Favart, L. Forthomme, A. Giammanco, J. Hollar, V. Lemaitre, J. Liao, O. Militaru, C. Nuttens, D. Pagano, A. Pin, K. Piotrzkowski, A. Popov, M. Selvaggi, J. M. Vizan Garcia, N. Beliy, T. Caebergs, E. Daubie, G. H. Hammad, G. A. Alves, M. Correa Martins Junior, T. Martins, M. E. Pol, M. H. G. Souza, W. L. Aldá Júnior, W. Carvalho, J. Chinellato, A. Custódio, E. M. Da Costa, D. De Jesus Damiao, C. De Oliveira Martins, S. Fonseca De Souza, H. Malbouisson, M. Malek, D. Matos Figueiredo, L. Mundim, H. Nogima, W. L. Prado Da Silva, A. Santoro, L. Soares Jorge, A. Sznajder, E. J. Tonelli Manganote, A. Vilela Pereira, T. S. Anjos, C. A. Bernardes, F. A. Dias, T. R. Fernandez Perez Tomei, E. M. Gregores, C. Lagana, F. Marinho, P. G. Mercadante, S. F. Novaes, Sandra S. Padula, V. Genchev, P. Iaydjiev, S. Piperov, M. Rodozov, S. Stoykova, G. Sultanov, V. Tcholakov, R. Trayanov, M. Vutova, A. Dimitrov, R. Hadjiiska, V. Kozhuharov, L. Litov, B. Pavlov, P. Petkov, J. G. Bian, G. M. Chen, H. S. Chen, C. H. Jiang, D. Liang, S. Liang, X. Meng, J. Tao, J. Wang, X. Wang, Z. Wang, H. Xiao, M. Xu, C. Asawatangtrakuldee, Y. Ban, Y. Guo, Q. Li, W. Li, S. Liu, Y. Mao, S. J. Qian, D. Wang, L. Zhang, W. Zou, C. Avila, C. A. Carrillo Montoya, J. P. Gomez, B. Gomez Moreno, J. C. Sanabria, N. Godinovic, D. Lelas, R. Plestina, D. Polic, I. Puljak, Z. Antunovic, M. Kovac, V. Brigljevic, S. Duric, K. Kadija, J. Luetic, D. Mekterovic, S. Morovic, L. Tikvica, A. Attikis, G. Mavromanolakis, J. Mousa, C. Nicolaou, F. Ptochos, P. A. Razis, M. Finger, M. Finger, Y. Assran, A. Ellithi Kamel, M. A. Mahmoud, A. Mahrous, A. Radi, M. Kadastik, M. Müntel, M. Murumaa, M. Raidal, L. Rebane, A. Tiko, P. Eerola, G. Fedi, M. Voutilainen, J. Härkönen, V. Karimäki, R. Kinnunen, M. J. Kortelainen, T. Lampén, K. Lassila-Perini, S. Lehti, T. Lindén, P. Luukka, T. Mäenpää, T. Peltola, E. Tuominen, J. Tuominiemi, E. Tuovinen, L. Wendland, A. Korpela, T. Tuuva, M. Besancon, S. Choudhury, F. Couderc, M. Dejardin, D. Denegri, B. Fabbro, J. L. Faure, F. Ferri, S. Ganjour, A. Givernaud, P. Gras, G. Hamel de Monchenault, P. Jarry, E. Locci, J. Malcles, L. Millischer, A. Nayak, J. Rander, A. Rosowsky, M. Titov, S. Baffioni, F. Beaudette, L. Benhabib, L. Bianchini, M. Bluj, P. Busson, C. Charlot, N. Daci, T. Dahms, M. Dalchenko, L. Dobrzynski, A. Florent, R. Granier de Cassagnac, M. Haguenauer, P. Miné, C. Mironov, I. N. Naranjo, M. Nguyen, C. Ochando, P. Paganini, D. Sabes, R. Salerno, Y. Sirois, C. Veelken, A. Zabi, J.-L. Agram, J. Andrea, D. Bloch, D. Bodin, J.-M. Brom, E. C. Chabert, C. Collard, E. Conte, F. Drouhin, J.-C. Fontaine, D. Gelé, U. Goerlach, C. Goetzmann, P. Juillot, A.-C. Le Bihan, P. Van Hove, S. Beauceron, N. Beaupere, G. Boudoul, S. Brochet, J. Chasserat, R. Chierici, D. Contardo, P. Depasse, H. El Mamouni, J. Fay, S. Gascon, M. Gouzevitch, B. Ille, T. Kurca, M. Lethuillier, L. Mirabito, S. Perries, L. Sgandurra, V. Sordini, Y. Tschudi, M. Vander Donckt, P. Verdier, S. Viret, Z. Tsamalaidze, C. Autermann, S. Beranek, B. Calpas, M. Edelhoff, L. Feld, N. Heracleous, O. Hindrichs, K. Klein, J. Merz, A. Ostapchuk, A. Perieanu, F. Raupach, J. Sammet, S. Schael, D. Sprenger, H. Weber, B. Wittmer, V. Zhukov, M. Ata, J. Caudron, E. Dietz-Laursonn, D. Duchardt, M. Erdmann, R. Fischer, A. Güth, T. Hebbeker, C. Heidemann, K. Hoepfner, D. Klingebiel, P. Kreuzer, M. Merschmeyer, A. Meyer, M. Olschewski, K. Padeken, P. Papacz, H. Pieta, H. Reithler, S. A. Schmitz, L. Sonnenschein, J. Steggemann, D. Teyssier, S. Thüer, M. Weber, V. Cherepanov, Y. Erdogan, G. Flügge, H. Geenen, M. Geisler, W. Haj Ahmad, F. Hoehle, B. Kargoll, T. Kress, Y. Kuessel, J. Lingemann, A. Nowack, I. M. Nugent, L. Perchalla, O. Pooth, A. Stahl, M. Aldaya Martin, I. Asin, N. Bartosik, J. Behr, W. Behrenhoff, U. Behrens, M. Bergholz, A. Bethani, K. Borras, A. Burgmeier, A. Cakir, L. Calligaris, A. Campbell, F. Costanza, D. Dammann, C. Diez Pardos, T. Dorland, G. Eckerlin, D. Eckstein, G. Flucke, A. Geiser, I. Glushkov, P. Gunnellini, S. Habib, J. Hauk, G. Hellwig, H. Jung, M. Kasemann, P. Katsas, C. Kleinwort, H. Kluge, M. Krämer, D. Krücker, E. Kuznetsova, W. Lange, J. Leonard, K. Lipka, W. Lohmann, B. Lutz, R. Mankel, I. Marfin, M. Marienfeld, I.-A. Melzer-Pellmann, A. B. Meyer, J. Mnich, A. Mussgiller, S. Naumann-Emme, O. Novgorodova, F. Nowak, J. Olzem, H. Perrey, A. Petrukhin, D. Pitzl, A. Raspereza, P. M. Ribeiro Cipriano, C. Riedl, E. Ron, M. Rosin, J. Salfeld-Nebgen, R. Schmidt, T. Schoerner-Sadenius, N. Sen, M. Stein, R. Walsh, C. Wissing, V. Blobel, H. Enderle, J. Erfle, U. Gebbert, M. Görner, M. Gosselink, J. Haller, K. Heine, R. S. Höing, G. Kaussen, H. Kirschenmann, R. Klanner, J. Lange, T. Peiffer, N. Pietsch, D. Rathjens, C. Sander, H. Schettler, P. Schleper, E. Schlieckau, A. Schmidt, M. Schröder, T. Schum, M. Seidel, J. Sibille, V. Sola, H. Stadie, G. Steinbrück, J. Thomsen, L. Vanelderen, C. Barth, C. Baus, J. Berger, C. Böser, T. Chwalek, W. De Boer, A. Descroix, A. Dierlamm, M. Feindt, M. Guthoff, C. Hackstein, F. Hartmann, T. Hauth, M. Heinrich, H. Held, K. H. Hoffmann, U. Husemann, I. Katkov, J. R. Komaragiri, A. Kornmayer, P. Lobelle Pardo, D. Martschei, S. Mueller, Th. Müller, M. Niegel, A. Nürnberg, O. Oberst, J. Ott, G. Quast, K. Rabbertz, F. Ratnikov, N. Ratnikova, S. Röcker, F.-P. Schilling, G. Schott, H. J. Simonis, F. M. Stober, D. Troendle, R. Ulrich, J. Wagner-Kuhr, S. Wayand, T. Weiler, M. Zeise, G. Anagnostou, G. Daskalakis, T. Geralis, S. Kesisoglou, A. Kyriakis, D. Loukas, A. Markou, C. Markou, E. Ntomari, L. Gouskos, T. J. Mertzimekis, A. Panagiotou, N. Saoulidou, E. Stiliaris, X. Aslanoglou, I. Evangelou, G. Flouris, C. Foudas, P. Kokkas, N. Manthos, I. Papadopoulos, E. Paradas, G. Bencze, C. Hajdu, P. Hidas, D. Horvath, B. Radics, F. Sikler, V. Veszpremi, G. Vesztergombi, A. J. Zsigmond, N. Beni, S. Czellar, J. Molnar, J. Palinkas, Z. Szillasi, J. Karancsi, P. Raics, Z. L. Trocsanyi, B. Ujvari, S. B. Beri, V. Bhatnagar, N. Dhingra, R. Gupta, M. Kaur, M. Z. Mehta, M. Mittal, N. Nishu, L. K. Saini, A. Sharma, J. B. Singh, Ashok Kumar, Arun Kumar, S. Ahuja, A. Bhardwaj, B. C. Choudhary, S. Malhotra, M. Naimuddin, K. Ranjan, P. Saxena, V. Sharma, R. K. Shivpuri, S. Banerjee, S. Bhattacharya, K. Chatterjee, S. Dutta, B. Gomber, Sa. Jain, Sh. Jain, R. Khurana, A. Modak, S. Mukherjee, D. Roy, S. Sarkar, M. Sharan, A. Abdulsalam, D. Dutta, S. Kailas, V. Kumar, A. K. Mohanty, L. M. Pant, P. Shukla, A. Topkar, T. Aziz, R. M. Chatterjee, S. Ganguly, M. Guchait, A. Gurtu, M. Maity, G. Majumder, K. Mazumdar, G. B. Mohanty, B. Parida, K. Sudhakar, N. Wickramage, S. Banerjee, S. Dugad, H. Arfaei, H. Bakhshiansohi, S. M. Etesami, A. Fahim, H. Hesari, A. Jafari, M. Khakzad, M. Mohammadi Najafabadi, S. Paktinat Mehdiabadi, B. Safarzadeh, M. Zeinali, M. Grunewald, M. Abbrescia, L. Barbone, C. Calabria, S. S. Chhibra, A. Colaleo, D. Creanza, N. De Filippis, M. De Palma, L. Fiore, G. Iaselli, G. Maggi, M. Maggi, B. Marangelli, S. My, S. Nuzzo, N. Pacifico, A. Pompili, G. Pugliese, G. Selvaggi, L. Silvestris, G. Singh, R. Venditti, P. Verwilligen, G. Zito, G. Abbiendi, A. C. Benvenuti, D. Bonacorsi, S. Braibant-Giacomelli, L. Brigliadori, R. Campanini, P. Capiluppi, A. Castro, F. R. Cavallo, M. Cuffiani, G. M. Dallavalle, F. Fabbri, A. Fanfani, D. Fasanella, P. Giacomelli, C. Grandi, L. Guiducci, S. Marcellini, G. Masetti, M. Meneghelli, A. Montanari, F. L. Navarria, F. Odorici, A. Perrotta, F. Primavera, A. M. Rossi, T. Rovelli, G. P. Siroli, N. Tosi, R. Travaglini, S. Albergo, M. Chiorboli, S. Costa, R. Potenza, A. Tricomi, C. Tuve, G. Barbagli, V. Ciulli, C. Civinini, R. D’Alessandro, E. Focardi, S. Frosali, E. Gallo, S. Gonzi, P. Lenzi, M. Meschini, S. Paoletti, G. Sguazzoni, A. Tropiano, L. Benussi, S. Bianco, F. Fabbri, D. Piccolo, P. Fabbricatore, R. Musenich, S. Tosi, A. Benaglia, F. De Guio, L. Di Matteo, S. Fiorendi, S. Gennai, A. Ghezzi, P. Govoni, M. T. Lucchini, S. Malvezzi, R. A. Manzoni, A. Martelli, A. Massironi, D. Menasce, L. Moroni, M. Paganoni, D. Pedrini, S. Ragazzi, N. Redaelli, T. Tabarelli de Fatis, S. Buontempo, N. Cavallo, A. De Cosa, F. Fabozzi, A. O. M. Iorio, L. Lista, S. Meola, M. Merola, P. Paolucci, P. Azzi, N. Bacchetta, P. Bellan, D. Bisello, A. Branca, R. Carlin, P. Checchia, T. Dorigo, M. Galanti, F. Gasparini, U. Gasparini, P. Giubilato, A. Gozzelino, K. Kanishchev, S. Lacaprara, I. Lazzizzera, M. Margoni, A. T. Meneguzzo, M. Michelotto, F. Montecassiano, M. Nespolo, J. Pazzini, M. Pegoraro, N. Pozzobon, P. Ronchese, F. Simonetto, E. Torassa, M. Tosi, P. Zotto, G. Zumerle, M. Gabusi, S. P. Ratti, C. Riccardi, P. Vitulo, M. Biasini, G. M. Bilei, L. Fanò, P. Lariccia, G. Mantovani, M. Menichelli, A. Nappi, F. Romeo, A. Saha, A. Santocchia, A. Spiezia, K. Androsov, P. Azzurri, G. Bagliesi, T. Boccali, G. Broccolo, R. Castaldi, R. T. D’Agnolo, R. Dell’Orso, F. Fiori, L. Foà, A. Giassi, A. Kraan, F. Ligabue, T. Lomtadze, L. Martini, A. Messineo, F. Palla, A. Rizzi, A. T. Serban, P. Spagnolo, P. Squillacioti, R. Tenchini, G. Tonelli, A. Venturi, P. G. Verdini, C. Vernieri, L. Barone, F. Cavallari, D. Del Re, M. Diemoz, C. Fanelli, M. Grassi, E. Longo, F. Margaroli, P. Meridiani, F. Micheli, S. Nourbakhsh, G. Organtini, R. Paramatti, S. Rahatlou, L. Soffi, N. Amapane, R. Arcidiacono, S. Argiro, M. Arneodo, C. Biino, N. Cartiglia, S. Casasso, M. Costa, P. De Remigis, N. Demaria, C. Mariotti, S. Maselli, E. Migliore, V. Monaco, M. Musich, M. M. Obertino, N. Pastrone, M. Pelliccioni, A. Potenza, A. Romero, M. Ruspa, R. Sacchi, A. Solano, A. Staiano, U. Tamponi, S. Belforte, V. Candelise, M. Casarsa, F. Cossutti, G. Della Ricca, B. Gobbo, C. La Licata, M. Marone, D. Montanino, A. Penzo, A. Schizzi, A. Zanetti, T. Y. Kim, S. K. Nam, S. Chang, D. H. Kim, G. N. Kim, J. E. Kim, D. J. Kong, Y. D. Oh, H. Park, D. C. Son, J. Y. Kim, Zero J. Kim, S. Song, S. Choi, D. Gyun, B. Hong, M. Jo, H. Kim, T. J. Kim, K. S. Lee, D. H. Moon, S. K. Park, Y. Roh, M. Choi, J. H. Kim, C. Park, I. C. Park, S. Park, G. Ryu, Y. Choi, Y. K. Choi, J. Goh, M. S. Kim, E. Kwon, B. Lee, J. Lee, S. Lee, H. Seo, I. Yu, I. Grigelionis, A. Juodagalvis, H. Castilla-Valdez, E. De La Cruz-Burelo, I. Heredia-de La Cruz, R. Lopez-Fernandez, J. Martínez-Ortega, A. Sanchez-Hernandez, L. M. Villasenor-Cendejas, S. Carrillo Moreno, F. Vazquez Valencia, H. A. Salazar Ibarguen, E. Casimiro Linares, A. Morelos Pineda, M. A. Reyes-Santos, D. Krofcheck, A. J. Bell, P. H. Butler, R. Doesburg, S. Reucroft, H. Silverwood, M. Ahmad, M. I. Asghar, J. Butt, H. R. Hoorani, S. Khalid, W. A. Khan, T. Khurshid, S. Qazi, M. A. Shah, M. Shoaib, H. Bialkowska, B. Boimska, T. Frueboes, M. Górski, M. Kazana, K. Nawrocki, K. Romanowska-Rybinska, M. Szleper, G. Wrochna, P. Zalewski, G. Brona, K. Bunkowski, M. Cwiok, W. Dominik, K. Doroba, A. Kalinowski, M. Konecki, J. Krolikowski, M. Misiura, W. Wolszczak, N. Almeida, P. Bargassa, A. David, P. Faccioli, P. G. Ferreira Parracho, M. Gallinaro, J. Seixas, J. Varela, P. Vischia, P. Bunin, M. Gavrilenko, I. Golutvin, I. Gorbunov, A. Kamenev, V. Karjavin, V. Konoplyanikov, G. Kozlov, A. Lanev, A. Malakhov, P. Moisenz, V. Palichik, V. Perelygin, S. Shmatov, V. Smirnov, A. Volodko, A. Zarubin, S. Evstyukhin, V. Golovtsov, Y. Ivanov, V. Kim, P. Levchenko, V. Murzin, V. Oreshkin, I. Smirnov, V. Sulimov, L. Uvarov, S. Vavilov, A. Vorobyev, An. Vorobyev, Yu. Andreev, A. Dermenev, S. Gninenko, N. Golubev, M. Kirsanov, N. Krasnikov, V. Matveev, A. Pashenkov, D. Tlisov, A. Toropin, V. Epshteyn, M. Erofeeva, V. Gavrilov, N. Lychkovskaya, V. Popov, G. Safronov, S. Semenov, A. Spiridonov, V. Stolin, E. Vlasov, A. Zhokin, V. Andreev, M. Azarkin, I. Dremin, M. Kirakosyan, A. Leonidov, G. Mesyats, S. V. Rusakov, A. Vinogradov, A. Belyaev, E. Boos, V. Bunichev, M. Dubinin, L. Dudko, A. Ershov, A. Gribushin, V. Klyukhin, I. Lokhtin, A. Markina, S. Obraztsov, M. Perfilov, V. Savrin, N. Tsirova, I. Azhgirey, I. Bayshev, S. Bitioukov, V. Kachanov, A. Kalinin, D. Konstantinov, V. Krychkine, V. Petrov, R. Ryutin, A. Sobol, L. Tourtchanovitch, S. Troshin, N. Tyurin, A. Uzunian, A. Volkov, P. Adzic, M. Ekmedzic, D. Krpic, J. Milosevic, M. Aguilar-Benitez, J. Alcaraz Maestre, C. Battilana, E. Calvo, M. Cerrada, M. Chamizo Llatas, N. Colino, B. De La Cruz, A. Delgado Peris, D. Domínguez Vázquez, C. Fernandez Bedoya, J. P. Fernández Ramos, A. Ferrando, J. Flix, M. C. Fouz, P. Garcia-Abia, O. Gonzalez Lopez, S. Goy Lopez, J. M. Hernandez, M. I. Josa, G. Merino, E. Navarro De Martino, J. Puerta Pelayo, A. Quintario Olmeda, I. Redondo, L. Romero, J. Santaolalla, M. S. Soares, C. Willmott, C. Albajar, J. F. de Trocóniz, H. Brun, J. Cuevas, J. Fernandez Menendez, S. Folgueras, I. Gonzalez Caballero, L. Lloret Iglesias, J. Piedra Gomez, J. A. Brochero Cifuentes, I. J. Cabrillo, A. Calderon, S. H. Chuang, J. Duarte Campderros, M. Fernandez, G. Gomez, J. Gonzalez Sanchez, A. Graziano, C. Jorda, A. Lopez Virto, J. Marco, R. Marco, C. Martinez Rivero, F. Matorras, F. J. Munoz Sanchez, T. Rodrigo, A. Y. Rodríguez-Marrero, A. Ruiz-Jimeno, L. Scodellaro, I. Vila, R. Vilar Cortabitarte, D. Abbaneo, E. Auffray, G. Auzinger, M. Bachtis, P. Baillon, A. H. Ball, D. Barney, J. Bendavid, J. F. Benitez, C. Bernet, G. Bianchi, P. Bloch, A. Bocci, A. Bonato, O. Bondu, C. Botta, H. Breuker, T. Camporesi, G. Cerminara, T. Christiansen, J. A. Coarasa Perez, S. Colafranceschi, D. d’Enterria, A. Dabrowski, A. De Roeck, S. De Visscher, S. Di Guida, M. Dobson, N. Dupont-Sagorin, A. Elliott-Peisert, J. Eugster, W. Funk, G. Georgiou, M. Giffels, D. Gigi, K. Gill, D. Giordano, M. Girone, M. Giunta, F. Glege, R. Gomez-Reino Garrido, S. Gowdy, R. Guida, J. Hammer, M. Hansen, P. Harris, C. Hartl, B. Hegner, A. Hinzmann, V. Innocente, P. Janot, K. Kaadze, E. Karavakis, K. Kousouris, K. Krajczar, P. Lecoq, Y.-J. Lee, C. Lourenço, N. Magini, M. Malberti, L. Malgeri, M. Mannelli, L. Masetti, F. Meijers, S. Mersi, E. Meschi, R. Moser, M. Mulders, P. Musella, E. Nesvold, L. Orsini, E. Palencia Cortezon, E. Perez, L. Perrozzi, A. Petrilli, A. Pfeiffer, M. Pierini, M. Pimiä, D. Piparo, G. Polese, L. Quertenmont, A. Racz, W. Reece, J. Rodrigues Antunes, G. Rolandi, C. Rovelli, M. Rovere, H. Sakulin, F. Santanastasio, C. Schäfer, C. Schwick, I. Segoni, S. Sekmen, A. Sharma, P. Siegrist, P. Silva, M. Simon, P. Sphicas, D. Spiga, M. Stoye, A. Tsirou, G. I. Veres, J. R. Vlimant, H. K. Wöhri, S. D. Worm, W. D. Zeuner, W. Bertl, K. Deiters, W. Erdmann, K. Gabathuler, R. Horisberger, Q. Ingram, H. C. Kaestli, S. König, D. Kotlinski, U. Langenegger, F. Meier, D. Renker, T. Rohe, F. Bachmair, L. Bäni, P. Bortignon, M. A. Buchmann, B. Casal, N. Chanon, A. Deisher, G. Dissertori, M. Dittmar, M. Donegà, M. Dünser, P. Eller, C. Grab, D. Hits, P. Lecomte, W. Lustermann, A. C. Marini, P. Martinez Ruiz del Arbol, N. Mohr, F. Moortgat, C. Nägeli, P. Nef, F. Nessi-Tedaldi, F. Pandolfi, L. Pape, F. Pauss, M. Peruzzi, F. J. Ronga, M. Rossini, L. Sala, A. K. Sanchez, A. Starodumov, B. Stieger, M. Takahashi, L. Tauscher, A. Thea, K. Theofilatos, D. Treille, C. Urscheler, R. Wallny, H. A. Weber, C. Amsler, V. Chiochia, C. Favaro, M. Ivova Rikova, B. Kilminster, B. Millan Mejias, P. Otiougova, P. Robmann, H. Snoek, S. Taroni, S. Tupputi, M. Verzetti, M. Cardaci, K. H. Chen, C. Ferro, C. M. Kuo, S. W. Li, W. Lin, Y. J. Lu, R. Volpe, S. S. Yu, P. Bartalini, P. Chang, Y. H. Chang, Y. W. Chang, Y. Chao, K. F. Chen, C. Dietz, U. Grundler, W.-S. Hou, Y. Hsiung, K. Y. Kao, Y. J. Lei, R.-S. Lu, D. Majumder, E. Petrakou, X. Shi, J. G. Shiu, Y. M. Tzeng, M. Wang, B. Asavapibhop, N. Suwonjandee, A. Adiguzel, M. N. Bakirci, S. Cerci, C. Dozen, I. Dumanoglu, E. Eskut, S. Girgis, G. Gokbulut, E. Gurpinar, I. Hos, E. E. Kangal, A. Kayis Topaksu, G. Onengut, K. Ozdemir, S. Ozturk, A. Polatoz, K. Sogut, D. Sunar Cerci, B. Tali, H. Topakli, M. Vergili, I. V. Akin, T. Aliev, B. Bilin, S. Bilmis, M. Deniz, H. Gamsizkan, A. M. Guler, G. Karapinar, K. Ocalan, A. Ozpineci, M. Serin, R. Sever, U. E. Surat, M. Yalvac, M. Zeyrek, E. Gülmez, B. Isildak, M. Kaya, O. Kaya, S. Ozkorucuklu, N. Sonmez, H. Bahtiyar, E. Barlas, K. Cankocak, Y. O. Günaydin, F. I. Vardarlı, M. Yücel, L. Levchuk, P. Sorokin, J. J. Brooke, E. Clement, D. Cussans, H. Flacher, R. Frazier, J. Goldstein, M. Grimes, G. P. Heath, H. F. Heath, L. Kreczko, S. Metson, D. M. Newbold, K. Nirunpong, A. Poll, S. Senkin, V. J. Smith, T. Williams, L. Basso, K. W. Bell, A. Belyaev, C. Brew, R. M. Brown, D. J. A. Cockerill, J. A. Coughlan, K. Harder, S. Harper, J. Jackson, E. Olaiya, D. Petyt, B. C. Radburn-Smith, C. H. Shepherd-Themistocleous, I. R. Tomalin, W. J. Womersley, R. Bainbridge, O. Buchmuller, D. Burton, D. Colling, N. Cripps, M. Cutajar, P. Dauncey, G. Davies, M. Della Negra, W. Ferguson, J. Fulcher, D. Futyan, A. Gilbert, A. Guneratne Bryer, G. Hall, Z. Hatherell, J. Hays, G. Iles, M. Jarvis, G. Karapostoli, M. Kenzie, R. Lane, R. Lucas, L. Lyons, A.-M. Magnan, J. Marrouche, B. Mathias, R. Nandi, J. Nash, A. Nikitenko, J. Pela, M. Pesaresi, K. Petridis, M. Pioppi, D. M. Raymond, S. Rogerson, A. Rose, C. Seez, P. Sharp, A. Sparrow, A. Tapper, M. Vazquez Acosta, T. Virdee, S. Wakefield, N. Wardle, T. Whyntie, M. Chadwick, J. E. Cole, P. R. Hobson, A. Khan, P. Kyberd, D. Leggat, D. Leslie, W. Martin, I. D. Reid, P. Symonds, L. Teodorescu, M. Turner, J. Dittmann, K. Hatakeyama, A. Kasmi, H. Liu, T. Scarborough, O. Charaf, S. I. Cooper, C. Henderson, P. Rumerio, A. Avetisyan, T. Bose, C. Fantasia, A. Heister, P. Lawson, D. Lazic, J. Rohlf, D. Sperka, J. St. John, L. Sulak, J. Alimena, S. Bhattacharya, G. Christopher, D. Cutts, Z. Demiragli, A. Ferapontov, A. Garabedian, U. Heintz, G. Kukartsev, E. Laird, G. Landsberg, M. Luk, M. Narain, M. Segala, T. Sinthuprasith, T. Speer, R. Breedon, G. Breto, M. Calderon De La Barca Sanchez, S. Chauhan, M. Chertok, J. Conway, R. Conway, P. T. Cox, R. Erbacher, M. Gardner, R. Houtz, W. Ko, A. Kopecky, R. Lander, O. Mall, T. Miceli, R. Nelson, D. Pellett, F. Ricci-Tam, B. Rutherford, M. Searle, J. Smith, M. Squires, M. Tripathi, R. Yohay, V. Andreev, D. Cline, R. Cousins, S. Erhan, P. Everaerts, C. Farrell, M. Felcini, J. Hauser, M. Ignatenko, C. Jarvis, G. Rakness, P. Schlein, P. Traczyk, V. Valuev, M. Weber, J. Babb, R. Clare, M. E. Dinardo, J. Ellison, J. W. Gary, F. Giordano, G. Hanson, H. Liu, O. R. Long, A. Luthra, H. Nguyen, S. Paramesvaran, J. Sturdy, S. Sumowidagdo, R. Wilken, S. Wimpenny, W. Andrews, J. G. Branson, G. B. Cerati, S. Cittolin, D. Evans, A. Holzner, R. Kelley, M. Lebourgeois, J. Letts, I. Macneill, B. Mangano, S. Padhi, C. Palmer, G. Petrucciani, M. Pieri, M. Sani, V. Sharma, S. Simon, E. Sudano, M. Tadel, Y. Tu, A. Vartak, S. Wasserbaech, F. Würthwein, A. Yagil, J. Yoo, D. Barge, R. Bellan, C. Campagnari, M. D’Alfonso, T. Danielson, A. Dishaw, K. Flowers, P. Geffert, C. George, F. Golf, J. Incandela, C. Justus, P. Kalavase, D. Kovalskyi, V. Krutelyov, S. Lowette, R. Magaña Villalba, N. Mccoll, V. Pavlunin, J. Ribnik, J. Richman, R. Rossin, D. Stuart, W. To, C. West, A. Apresyan, A. Bornheim, J. Bunn, Y. Chen, E. Di Marco, J. Duarte, D. Kcira, Y. Ma, A. Mott, H. B. Newman, C. Rogan, M. Spiropulu, V. Timciuc, J. Veverka, R. Wilkinson, S. Xie, Y. Yang, R. Y. Zhu, V. Azzolini, A. Calamba, R. Carroll, T. Ferguson, Y. Iiyama, D. W. Jang, Y. F. Liu, M. Paulini, J. Russ, H. Vogel, I. Vorobiev, J. P. Cumalat, B. R. Drell, W. T. Ford, A. Gaz, E. Luiggi Lopez, U. Nauenberg, J. G. Smith, K. Stenson, K. A. Ulmer, S. R. Wagner, J. Alexander, A. Chatterjee, N. Eggert, L. K. Gibbons, W. Hopkins, A. Khukhunaishvili, B. Kreis, N. Mirman, B. Nachman, G. Nicolas Kaufman, J. R. Patterson, A. Ryd, E. Salvati, W. Sun, W. D. Teo, J. Thom, J. Thompson, J. Tucker, Y. Weng, L. Winstrom, P. Wittich, D. Winn, S. Abdullin, M. Albrow, J. Anderson, G. Apollinari, L. A. T. Bauerdick, A. Beretvas, J. Berryhill, P. C. Bhat, K. Burkett, J. N. Butler, V. Chetluru, H. W. K. Cheung, F. Chlebana, S. Cihangir, V. D. Elvira, I. Fisk, J. Freeman, Y. Gao, E. Gottschalk, L. Gray, D. Green, O. Gutsche, R. M. Harris, J. Hirschauer, B. Hooberman, S. Jindariani, M. Johnson, U. Joshi, B. Klima, S. Kunori, S. Kwan, J. Linacre, D. Lincoln, R. Lipton, J. Lykken, K. Maeshima, J. M. Marraffino, V. I. Martinez Outschoorn, S. Maruyama, D. Mason, P. McBride, K. Mishra, S. Mrenna, Y. Musienko, C. Newman-Holmes, V. O’Dell, O. Prokofyev, E. Sexton-Kennedy, S. Sharma, W. J. Spalding, L. Spiegel, L. Taylor, S. Tkaczyk, N. V. Tran, L. Uplegger, E. W. Vaandering, R. Vidal, J. Whitmore, W. Wu, F. Yang, J. C. Yun, D. Acosta, P. Avery, D. Bourilkov, M. Chen, T. Cheng, S. Das, M. De Gruttola, G. P. Di Giovanni, D. Dobur, A. Drozdetskiy, R. D. Field, M. Fisher, Y. Fu, I. K. Furic, J. Hugon, B. Kim, J. Konigsberg, A. Korytov, A. Kropivnitskaya, T. Kypreos, J. F. Low, K. Matchev, P. Milenovic, G. Mitselmakher, L. Muniz, R. Remington, A. Rinkevicius, N. Skhirtladze, M. Snowball, J. Yelton, M. Zakaria, V. Gaultney, S. Hewamanage, L. M. Lebolo, S. Linn, P. Markowitz, G. Martinez, J. L. Rodriguez, T. Adams, A. Askew, J. Bochenek, J. Chen, B. Diamond, S. V. Gleyzer, J. Haas, S. Hagopian, V. Hagopian, K. F. Johnson, H. Prosper, V. Veeraraghavan, M. Weinberg, M. M. Baarmand, B. Dorney, M. Hohlmann, H. Kalakhety, F. Yumiceva, M. R. Adams, L. Apanasevich, V. E. Bazterra, R. R. Betts, I. Bucinskaite, J. Callner, R. Cavanaugh, O. Evdokimov, L. Gauthier, C. E. Gerber, D. J. Hofman, S. Khalatyan, P. Kurt, F. Lacroix, C. O’Brien, C. Silkworth, D. Strom, P. Turner, N. Varelas, U. Akgun, E. A. Albayrak, B. Bilki, W. Clarida, K. Dilsiz, F. Duru, S. Griffiths, J.-P. Merlo, H. Mermerkaya, A. Mestvirishvili, A. Moeller, J. Nachtman, C. R. Newsom, H. Ogul, Y. Onel, F. Ozok, S. Sen, P. Tan, E. Tiras, J. Wetzel, T. Yetkin, K. Yi, B. A. Barnett, B. Blumenfeld, S. Bolognesi, D. Fehling, G. Giurgiu, A. V. Gritsan, G. Hu, P. Maksimovic, M. Swartz, A. Whitbeck, P. Baringer, A. Bean, G. Benelli, R. P. Kenny III, M. Murray, D. Noonan, S. Sanders, R. Stringer, J. S. Wood, A. F. Barfuss, I. Chakaberia, A. Ivanov, S. Khalil, M. Makouski, Y. Maravin, S. Shrestha, I. Svintradze, J. Gronberg, D. Lange, F. Rebassoo, D. Wright, A. Baden, B. Calvert, S. C. Eno, J. A. Gomez, N. J. Hadley, R. G. Kellogg, T. Kolberg, Y. Lu, M. Marionneau, A. C. Mignerey, K. Pedro, A. Peterman, A. Skuja, J. Temple, M. B. Tonjes, S. C. Tonwar, A. Apyan, G. Bauer, W. Busza, E. Butz, I. A. Cali, M. Chan, V. Dutta, G. Gomez Ceballos, M. Goncharov, Y. Kim, M. Klute, Y. S. Lai, A. Levin, P. D. Luckey, T. Ma, S. Nahn, C. Paus, D. Ralph, C. Roland, G. Roland, G. S. F. Stephans, F. Stöckli, K. Sumorok, K. Sung, D. Velicanu, R. Wolf, B. Wyslouch, M. Yang, Y. Yilmaz, A. S. Yoon, M. Zanetti, V. Zhukova, B. Dahmes, A. De Benedetti, G. Franzoni, A. Gude, J. Haupt, S. C. Kao, K. Klapoetke, Y. Kubota, J. Mans, N. Pastika, R. Rusack, M. Sasseville, A. Singovsky, N. Tambe, J. Turkewitz, L. M. Cremaldi, R. Kroeger, L. Perera, R. Rahmat, D. A. Sanders, D. Summers, E. Avdeeva, K. Bloom, S. Bose, D. R. Claes, A. Dominguez, M. Eads, R. Gonzalez Suarez, J. Keller, I. Kravchenko, J. Lazo-Flores, S. Malik, G. R. Snow, J. Dolen, A. Godshalk, I. Iashvili, S. Jain, A. Kharchilava, A. Kumar, S. Rappoccio, Z. Wan, G. Alverson, E. Barberis, D. Baumgartel, M. Chasco, J. Haley, D. Nash, T. Orimoto, D. Trocino, D. Wood, J. Zhang, A. Anastassov, K. A. Hahn, A. Kubik, L. Lusito, N. Mucia, N. Odell, B. Pollack, A. Pozdnyakov, M. Schmitt, S. Stoynev, M. Velasco, S. Won, D. Berry, A. Brinkerhoff, K. M. Chan, M. Hildreth, C. Jessop, D. J. Karmgard, J. Kolb, K. Lannon, W. Luo, S. Lynch, N. Marinelli, D. M. Morse, T. Pearson, M. Planer, R. Ruchti, J. Slaunwhite, N. Valls, M. Wayne, M. Wolf, L. Antonelli, B. Bylsma, L. S. Durkin, C. Hill, R. Hughes, K. Kotov, T. Y. Ling, D. Puigh, M. Rodenburg, G. Smith, C. Vuosalo, G. Williams, B. L. Winer, H. Wolfe, E. Berry, P. Elmer, V. Halyo, P. Hebda, J. Hegeman, A. Hunt, P. Jindal, S. A. Koay, D. Lopes Pegna, P. Lujan, D. Marlow, T. Medvedeva, M. Mooney, J. Olsen, P. Piroué, X. Quan, A. Raval, H. Saka, D. Stickland, C. Tully, J. S. Werner, S. C. Zenz, A. Zuranski, E. Brownson, A. Lopez, H. Mendez, J. E. Ramirez Vargas, E. Alagoz, D. Benedetti, G. Bolla, D. Bortoletto, M. De Mattia, A. Everett, Z. Hu, M. Jones, O. Koybasi, M. Kress, N. Leonardo, V. Maroussov, P. Merkel, D. H. Miller, N. Neumeister, I. Shipsey, D. Silvers, A. Svyatkovskiy, M. Vidal Marono, H. D. Yoo, J. Zablocki, Y. Zheng, S. Guragain, N. Parashar, A. Adair, B. Akgun, K. M. Ecklund, F. J. M. Geurts, W. Li, B. P. Padley, R. Redjimi, J. Roberts, J. Zabel, B. Betchart, A. Bodek, R. Covarelli, P. de Barbaro, R. Demina, Y. Eshaq, T. Ferbel, A. Garcia-Bellido, P. Goldenzweig, J. Han, A. Harel, D. C. Miner, G. Petrillo, D. Vishnevskiy, M. Zielinski, A. Bhatti, R. Ciesielski, L. Demortier, K. Goulianos, G. Lungu, S. Malik, C. Mesropian, S. Arora, A. Barker, J. P. Chou, C. Contreras-Campana, E. Contreras-Campana, D. Duggan, D. Ferencek, Y. Gershtein, R. Gray, E. Halkiadakis, D. Hidas, A. Lath, S. Panwalkar, M. Park, R. Patel, V. Rekovic, J. Robles, K. Rose, S. Salur, S. Schnetzer, C. Seitz, S. Somalwar, R. Stone, M. Walker, G. Cerizza, M. Hollingsworth, S. Spanier, Z. C. Yang, A. York, R. Eusebi, W. Flanagan, J. Gilmore, T. Kamon, V. Khotilovich, R. Montalvo, I. Osipenkov, Y. Pakhotin, A. Perloff, J. Roe, A. Safonov, T. Sakuma, I. Suarez, A. Tatarinov, D. Toback, N. Akchurin, J. Damgov, C. Dragoiu, P. R. Dudero, C. Jeong, K. Kovitanggoon, S. W. Lee, T. Libeiro, I. Volobouev, E. Appelt, A. G. Delannoy, S. Greene, A. Gurrola, W. Johns, C. Maguire, Y. Mao, A. Melo, M. Sharma, P. Sheldon, B. Snook, S. Tuo, J. Velkovska, M. W. Arenton, M. Balazs, S. Boutle, B. Cox, B. Francis, J. Goodell, R. Hirosky, A. Ledovskoy, C. Lin, C. Neu, J. Wood, S. Gollapinni, R. Harr, P. E. Karchin, C. Kottachchi Kankanamge Don, P. Lamichhane, A. Sakharov, M. Anderson, D. A. Belknap, L. Borrello, D. Carlsmith, M. Cepeda, S. Dasu, E. Friis, K. S. Grogg, M. Grothe, R. Hall-Wilton, M. Herndon, A. Hervé, P. Klabbers, J. Klukas, A. Lanaro, C. Lazaridis, R. Loveless, A. Mohapatra, M. U. Mozer, I. Ojalvo, G. A. Pierro, I. Ross, A. Savin, W. H. Smith, J. Swanson

**Affiliations:** 1 CERN, Geneva, Switzerland; 2 Yerevan Physics Institute, Yerevan, Armenia; 3 Institut für Hochenergiephysik der OeAW, Wien, Austria; 4 National Centre for Particle and High Energy Physics, Minsk, Belarus; 5 Universiteit Antwerpen, Antwerpen, Belgium; 6 Vrije Universiteit Brussel, Brussel, Belgium; 7 Université Libre de Bruxelles, Bruxelles, Belgium; 8 Ghent University, Ghent, Belgium; 9 Université Catholique de Louvain, Louvain-la-Neuve, Belgium; 10 Université de Mons, Mons, Belgium; 11 Centro Brasileiro de Pesquisas Fisicas, Rio de Janeiro, Brazil; 12 Universidade do Estado do Rio de Janeiro, Rio de Janeiro, Brazil; 13 Universidade Estadual Paulista, São Paulo, Brazil; 14 Universidade Federal do ABC, São Paulo, Brazil; 15 Institute for Nuclear Research and Nuclear Energy, Sofia, Bulgaria; 16 University of Sofia, Sofia, Bulgaria; 17 Institute of High Energy Physics, Beijing, China; 18 State Key Laboratory of Nuclear Physics and Technology, Peking University, Beijing, China; 19 Universidad de Los Andes, Bogota, Colombia; 20 Technical University of Split, Split, Croatia; 21 University of Split, Split, Croatia; 22 Institute Rudjer Boskovic, Zagreb, Croatia; 23 University of Cyprus, Nicosia, Cyprus; 24 Charles University, Prague, Czech Republic; 25 Academy of Scientific Research and Technology of the Arab Republic of Egypt, Egyptian Network of High Energy Physics, Cairo, Egypt; 26 National Institute of Chemical Physics and Biophysics, Tallinn, Estonia; 27 Department of Physics, University of Helsinki, Helsinki, Finland; 28 Helsinki Institute of Physics, Helsinki, Finland; 29 Lappeenranta University of Technology, Lappeenranta, Finland; 30 DSM/IRFU, CEA/Saclay, Gif-sur-Yvette, France; 31 Laboratoire Leprince-Ringuet, Ecole Polytechnique, IN2P3-CNRS, Palaiseau, France; 32 Institut Pluridisciplinaire Hubert Curien, Université de Strasbourg, Université de Haute Alsace Mulhouse, CNRS/IN2P3, Strasbourg, France; 33 Université de Lyon, Université Claude Bernard Lyon 1, Institut de Physique Nucléaire de Lyon, CNRS-IN2P3, Villeurbanne, France; 34 Institute of High Energy Physics and Informatization, Tbilisi State University, Tbilisi, Georgia; 35 I. Physikalisches Institut, RWTH Aachen University, Aachen, Germany; 36 III. Physikalisches Institut A, RWTH Aachen University, Aachen, Germany; 37 III. Physikalisches Institut B, RWTH Aachen University, Aachen, Germany; 38 Deutsches Elektronen-Synchrotron, Hamburg, Germany; 39 University of Hamburg, Hamburg, Germany; 40 Institut für Experimentelle Kernphysik, Karlsruhe, Germany; 41 Institute of Nuclear and Particle Physics (INPP), NCSR Demokritos, Aghia Paraskevi, Greece; 42 University of Athens, Athens, Greece; 43 University of Ioánnina, Ioánnina, Greece; 44 KFKI Research Institute for Particle and Nuclear Physics, Budapest, Hungary; 45 Institute of Nuclear Research ATOMKI, Debrecen, Hungary; 46 University of Debrecen, Debrecen, Hungary; 47 Panjab University, Chandigarh, India; 48 University of Delhi, Delhi, India; 49 Saha Institute of Nuclear Physics, Kolkata, India; 50 Bhabha Atomic Research Centre, Mumbai, India; 51 Tata Institute of Fundamental Research - EHEP, Mumbai, India; 52 Tata Institute of Fundamental Research - HECR, Mumbai, India; 53 Institute for Research in Fundamental Sciences (IPM), Tehran, Iran; 54 University College Dublin, Dublin, Ireland; 55 INFN Sezione di Bari, Bari, Italy; 56 Università di Bari, Bari, Italy; 57 Politecnico di Bari, Bari, Italy; 58 INFN Sezione di Bologna, Bologna, Italy; 59 Università di Bologna, Bologna, Italy; 60 INFN Sezione di Catania, Catania, Italy; 61 Università di Catania, Catania, Italy; 62 INFN Sezione di Firenze, Firenze, Italy; 63 Università di Firenze, Firenze, Italy; 64 INFN Laboratori Nazionali di Frascati, Frascati, Italy; 65 INFN Sezione di Genova, Genova, Italy; 66 Università di Genova, Genova, Italy; 67 INFN Sezione di Milano-Bicocca, Milano, Italy; 68 Università di Milano-Bicocca, Milano, Italy; 69 INFN Sezione di Napoli, Napoli, Italy; 70 Università di Napoli ’Federico II’, Napoli, Italy; 71 Università della Basilicata (Potenza), Napoli, Italy; 72 Università G. Marconi (Roma), Napoli, Italy; 73 INFN Sezione di Padova, Padova, Italy; 74 Università di Padova, Padova, Italy; 75 Università di Trento (Trento), Padova, Italy; 76 INFN Sezione di Pavia, Pavia, Italy; 77 Università di Pavia, Pavia, Italy; 78 INFN Sezione di Perugia, Perugia, Italy; 79 Università di Perugia, Perugia, Italy; 80 INFN Sezione di Pisa, Pisa, Italy; 81 Università di Pisa, Pisa, Italy; 82 Scuola Normale Superiore di Pisa, Pisa, Italy; 83 INFN Sezione di Roma, Roma, Italy; 84 Università di Roma, Roma, Italy; 85 INFN Sezione di Torino, Torino, Italy; 86 Università di Torino, Torino, Italy; 87 Università del Piemonte Orientale (Novara), Torino, Italy; 88 INFN Sezione di Trieste, Trieste, Italy; 89 Università di Trieste, Trieste, Italy; 90 Kangwon National University, Chunchon, Korea; 91 Kyungpook National University, Daegu, Korea; 92 Institute for Universe and Elementary Particles, Chonnam National University, Kwangju, Korea; 93 Korea University, Seoul, Korea; 94 University of Seoul, Seoul, Korea; 95 Sungkyunkwan University, Suwon, Korea; 96 Vilnius University, Vilnius, Lithuania; 97 Centro de Investigacion y de Estudios Avanzados del IPN, Mexico City, Mexico; 98 Universidad Iberoamericana, Mexico City, Mexico; 99 Benemerita Universidad Autonoma de Puebla, Puebla, Mexico; 100 Universidad Autónoma de San Luis Potosí, San Luis Potosí, Mexico; 101 University of Auckland, Auckland, New Zealand; 102 University of Canterbury, Christchurch, New Zealand; 103 National Centre for Physics, Quaid-I-Azam University, Islamabad, Pakistan; 104 National Centre for Nuclear Research, Swierk, Poland; 105 Institute of Experimental Physics, Faculty of Physics, University of Warsaw, Warsaw, Poland; 106 Laboratório de Instrumentação e Física Experimental de Partículas, Lisboa, Portugal; 107 Joint Institute for Nuclear Research, Dubna, Russia; 108 Petersburg Nuclear Physics Institute, Gatchina (St. Petersburg), Russia; 109 Institute for Nuclear Research, Moscow, Russia; 110 Institute for Theoretical and Experimental Physics, Moscow, Russia; 111 P.N. Lebedev Physical Institute, Moscow, Russia; 112 Skobeltsyn Institute of Nuclear Physics, Lomonosov Moscow State University, Moscow, Russia; 113 State Research Center of Russian Federation, Institute for High Energy Physics, Protvino, Russia; 114 Faculty of Physics and Vinca Institute of Nuclear Sciences, University of Belgrade, Belgrade, Serbia; 115 Centro de Investigaciones Energéticas Medioambientales y Tecnológicas (CIEMAT), Madrid, Spain; 116 Universidad Autónoma de Madrid, Madrid, Spain; 117 Universidad de Oviedo, Oviedo, Spain; 118 Instituto de Física de Cantabria (IFCA), CSIC-Universidad de Cantabria, Santander, Spain; 119 European Organization for Nuclear Research, CERN, Geneva, Switzerland; 120 Paul Scherrer Institut, Villigen, Switzerland; 121 Institute for Particle Physics, ETH Zurich, Zurich, Switzerland; 122 Universität Zürich, Zurich, Switzerland; 123 National Central University, Chung-Li, Taiwan; 124 National Taiwan University (NTU), Taipei, Taiwan; 125 Chulalongkorn University, Bangkok, Thailand; 126 Cukurova University, Adana, Turkey; 127 Physics Department, Middle East Technical University, Ankara, Turkey; 128 Bogazici University, Istanbul, Turkey; 129 Istanbul Technical University, Istanbul, Turkey; 130 National Scientific Center, Kharkov Institute of Physics and Technology, Kharkov, Ukraine; 131 University of Bristol, Bristol, United Kingdom; 132 Rutherford Appleton Laboratory, Didcot, United Kingdom; 133 Imperial College, London, United Kingdom; 134 Brunel University, Uxbridge, United Kingdom; 135 Baylor University, Waco, USA; 136 The University of Alabama, Tuscaloosa, USA; 137 Boston University, Boston, USA; 138 Brown University, Providence, USA; 139 University of California, Davis, Davis, USA; 140 University of California, Los Angeles, USA; 141 University of California, Riverside, Riverside, USA; 142 University of California, San Diego, La Jolla, USA; 143 University of California, Santa Barbara, Santa Barbara, USA; 144 California Institute of Technology, Pasadena, USA; 145 Carnegie Mellon University, Pittsburgh, USA; 146 University of Colorado at Boulder, Boulder, USA; 147 Cornell University, Ithaca, USA; 148 Fairfield University, Fairfield, USA; 149 Fermi National Accelerator Laboratory, Batavia, USA; 150 University of Florida, Gainesville, USA; 151 Florida International University, Miami, USA; 152 Florida State University, Tallahassee, USA; 153 Florida Institute of Technology, Melbourne, USA; 154 University of Illinois at Chicago (UIC), Chicago, USA; 155 The University of Iowa, Iowa City, USA; 156 Johns Hopkins University, Baltimore, USA; 157 The University of Kansas, Lawrence, USA; 158 Kansas State University, Manhattan, USA; 159 Lawrence Livermore National Laboratory, Livermore, USA; 160 University of Maryland, College Park, USA; 161 Massachusetts Institute of Technology, Cambridge, USA; 162 University of Minnesota, Minneapolis, USA; 163 University of Mississippi, Oxford, USA; 164 University of Nebraska-Lincoln, Lincoln, USA; 165 State University of New York at Buffalo, Buffalo, USA; 166 Northeastern University, Boston, USA; 167 Northwestern University, Evanston, USA; 168 University of Notre Dame, Notre Dame, USA; 169 The Ohio State University, Columbus, USA; 170 Princeton University, Princeton, USA; 171 University of Puerto Rico, Mayaguez, USA; 172 Purdue University, West Lafayette, USA; 173 Purdue University Calumet, Hammond, USA; 174 Rice University, Houston, USA; 175 University of Rochester, Rochester, USA; 176 The Rockefeller University, New York, USA; 177 Rutgers, The State University of New Jersey, Piscataway, USA; 178 University of Tennessee, Knoxville, USA; 179 Texas A&M University, College Station, USA; 180 Texas Tech University, Lubbock, USA; 181 Vanderbilt University, Nashville, USA; 182 University of Virginia, Charlottesville, USA; 183 Wayne State University, Detroit, USA; 184 University of Wisconsin, Madison, USA

## Abstract

A simultaneous measurement of the top-quark, W-boson, and neutrino masses is reported for $\mathrm{t}\overline{\mathrm{t}} $ events selected in the dilepton final state from a data sample corresponding to an integrated luminosity of 5.0 fb^−1^ collected by the CMS experiment in pp collisions at $\sqrt{s}=7 ~\text{TeV} $. The analysis is based on endpoint determinations in kinematic distributions. When the neutrino and W-boson masses are constrained to their world-average values, a top-quark mass value of $M_{\mathrm{t}}=173.9\pm0.9 ~\text{(stat.)} {}^{+1.7}_{-2.1} ~\text{(syst.)} ~\text{GeV} $ is obtained. When such constraints are not used, the three particle masses are obtained in a simultaneous fit. In this unconstrained mode the study serves as a test of mass determination methods that may be used in beyond standard model physics scenarios where several masses in a decay chain may be unknown and undetected particles lead to underconstrained kinematics.

## Introduction

The determination of the top-quark mass sets a fundamental benchmark for the standard model (SM), and is one of the precision measurements that defines electroweak constraints on possible new physics beyond the SM [1]. With the recent observations [2, 3] of a Higgs boson candidate at a mass of approximately 125 GeV, existing data can now overconstrain the SM. The top quark plays an important role in such constraints because its large mass, appearing quadratically in loop corrections to many SM observables, dominates other contributions. It is also key to the quartic term in the Higgs potential at high energy, and therefore to the question of stability of the electroweak vacuum [4, 5]. For these reasons, precise top-quark mass determinations are essential to characterize and probe the SM. Recent results obtained at the Large Hadron Collider (LHC) for the top-quark mass in $\mathrm{t}\overline{\mathrm{t}} $ events include those reported by ATLAS [6], *M*_t_=174.5±0.6 (stat.)±2.3 (syst.) GeV, and by the Compact Muon Solenoid (CMS) [7], *M*_t_=173.49±0.43 (stat.)±0.98 (syst.) GeV, using the semileptonic decay channel of the $\mathrm {t}\overline{\mathrm{t}}$ pair. The CMS Collaboration has also reported a measurement [8] in the dilepton channel, *M*_t_=172.5±0.4 (stat.)±1.5 (syst.) GeV. A recent summary of top-quark mass measurements conducted by the CDF and D0 Collaborations [9] reports a combined result *M*_t_=173.18±0.56 (stat.)±0.75 (syst.) GeV.

In parallel with recent measurements of the properties of the top quark at the LHC, there has been a great deal of theoretical progress on methods using endpoints of kinematic variables to measure particle masses with minimal input from simulation. These methods are generally aimed at measuring the masses of new particles, should they be discovered, but can also be applied to measure the masses of standard model particles such as the top quark. Such an application acts as both a test of the methods and a measurement of the top-quark mass utilizing technique very different from those used in previous studies.

Indeed, top-quark pair production provides a good match to these new methods, as dilepton decays of top-quark pairs ($\mathrm {t}\overline{\mathrm {t}}\to (\mathrm {b}\ell^{+}\nu)(\overline{\mathrm {b}}\ell ^{-}\bar{\nu})$) provide challenges in mass measurement very similar to the ones that these methods were designed to solve. A key feature of many current theories of physics beyond the standard model is the existence of a candidate for dark matter, such as a weakly interacting massive particle (WIMP). These particles are usually stabilized in a theory by a conserved parity, often introduced *ad hoc*, under which SM particles are even and new-physics particles are odd. Examples include *R*-parity in supersymmetry (SUSY) and *T*-parity in little-Higgs models. One consequence of this parity is that new physics particles must be produced in pairs. Each of the pair-produced particles will then decay to a cascade of SM particles, terminating with the lightest odd-parity particle of the new theory. In such cases, there will be two particles which do not interact with the detector, yielding events where the observable kinematics are underconstrained. Mass measurements in these events are further complicated by the presence of multiple new particles with unknown masses.

**Fig. 1:**
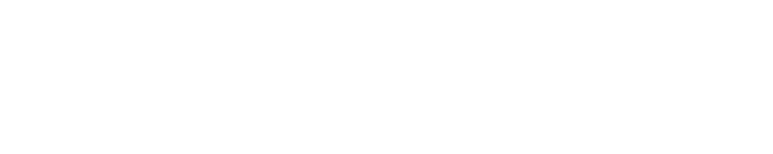
Top-quark pair dilepton decays, with two jets, two leptons, and two unobserved particles (*left*) exhibit a signature similar to some SUSY modes (*right*). In the figure, $\widetilde{u}$, $\widetilde{\chi}^{\pm}$, $\widetilde{\nu}$, and $\widetilde{\chi}^{0} $ denote the u-squark, chargino, sneutrino, and neutralino respectively; *an asterisk* indicates the antiparticle of the corresponding SUSY particle

The dilepton decays of $\mathrm{t}\overline{\mathrm {t}}$ events at the LHC offer a rich source of symmetric decay chains terminating in two neutrinos. With their combination of jets, leptons, and undetected particles, these $\mathrm{t}\overline{\mathrm{t}}$ events bear close kinematic and topological resemblance to new-physics scenarios such as the supersymmetric decay chain illustrated in Fig. 1. This correspondence has motivated [10] the idea to use the abundant $\mathrm{t}\overline{\mathrm{t}}$ samples of the LHC as a testbed for the new methods and novel observables that have been proposed to handle mass measurement in new-physics events [11]. A simultaneous measurement of the top-quark, W-boson, and neutrino masses in dilepton $\mathrm{t}\overline{\mathrm{t}}$ decays closely mimics the strategies needed for studies of new physics.

The analysis presented here focuses on the *M*_T2_ variable and its variants [11, 12]. These kinematic observables are mass estimators that will be defined in Sect. 4. The goals of this analysis are two-fold: to demonstrate the performance of a new mass measurement technique, and to make a precise measurement of the top-quark mass. To demonstrate the performance of the method, we apply it to the $\mathrm{t}\overline{\mathrm {t}}$ system assuming no knowledge of the W-boson or neutrino masses. This allows us to measure the masses of all three undetected particles involved in the dilepton decay: the top quark, W boson, and neutrino. This “unconstrained” fit provides a test of the method under conditions similar to what one might expect to find when attempting to measure the masses of new particles. In order to make a precise measurement of the top-quark mass, on the other hand, we assume the world-average values for the W boson and neutrino masses. This “doubly-constrained” fit achieves a precision in the top-quark mass determination similar to that obtained by traditional methods. The *M*_T2_ observable has been previously suggested [13] or used [14] in top-quark mass measurements.

In considering any top-quark mass measurement, however, it is critical to confront the fact that deep theoretical problems complicate the interpretation of the measurement. The issues arise because a top quark is a colored object while the W boson and hadronic jet observed in the final state are not. In the transition t→Wb, a single color charge must come from elsewhere to neutralize the final-state b jet, with the inevitable consequence that the observed energy and momentum of the final state differ from that of the original top quark. The resulting difference between measured mass and top-quark mass is therefore at least at the level at which soft color exchanges occur, i.e. ∼*Λ*_QCD_ [15, 16]. In the current state of the art, a Monte Carlo (MC) generator is normally used to fix a relationship between the experimentally measured mass of the final state and a top-quark mass parameter of the simulation; but model assumptions upon which the simulation of nonperturbative physics depend further limit the precision of such interpretative statements to about 1 GeV [17].

We therefore take care in this measurement to distinguish between the interpretive use of MC simulation described above, which is inherently model dependent, and experimental procedures, which can be made clear and model independent. A distinctive feature of the top-quark mass measurement reported here is its limited dependence on MC simulation. There is no reliance on MC templates [14], and the endpoint method gives a result which is consistent with the kinematic mass in MC without further tuning or correction. For this reason, the measurement outlined here complements the set of conventional top-quark mass measurements, and is applicable to new-physics scenarios where MC simulation is used sparingly.

## The CMS detector and event reconstruction

The central feature of the CMS apparatus is a superconducting solenoid of 6 m internal diameter, providing a magnetic field of 3.8 T. Inside the superconducting solenoid volume are silicon pixel and strip trackers, a lead tungstate crystal electromagnetic calorimeter, and a brass/scintillator hadron calorimeter. Muons are measured in gas-ionization detectors embedded in the steel flux return yoke. Extensive forward calorimetry complements the coverage provided by the barrel and endcap detectors. A more detailed description of the CMS detector can be found in Ref. [18].

Jets, electrons, muons, and missing transverse momentum are reconstructed using a global event reconstruction technique, also called particle-flow event reconstruction [19, 20]. Hadronic jets are clustered from the reconstructed particles with the infrared and collinear-safe anti-*k*_T_ algorithm [21], using a size parameter 0.5. The jet momentum is determined as the vectorial sum of all particle momenta in this jet, and is found in the simulation to be within 5 % to 10 % of the true momentum over the whole transverse momentum (*p*_T_) spectrum and detector acceptance. Jet energy corrections are derived from the simulation, and are confirmed in measurements on data with the energy balance of dijet and photon + jet events [22]. The jet energy resolution amounts typically to 15 % at jet *p*_T_ of 10 GeV, 8 % at 100 GeV, and 4 % at 1 TeV. The missing transverse momentum vector is defined by  where the sum is taken over all particle-flow objects in the event; and missing transverse “energy” is given by .

## Event selection

The data set used for this analysis corresponds to an integrated luminosity of 5.0 fb^−1^ of proton-proton collisions at $\sqrt{s}=7 ~\text {TeV} $ recorded by the CMS detector in 2011. We apply an event selection to isolate a dilepton sample that is largely free of backgrounds. We require two well-identified and isolated opposite-sign leptons (electrons or muons) passing dilepton trigger requirements; the minimum *p*_T_ requirements for the triggers are 17 GeV and 8 GeV for the leading and sub-leading leptons. In addition we require at least two b-tagged jets, subsequently used in the top-quark reconstruction, and missing transverse energy. Here and throughout this paper, we use *ℓ* (and “lepton”) to denote an electron or muon; the signal decays of interest are t→b*ℓν*. Leptons must satisfy *p*_T_>20 GeV and the event is vetoed if the leptons have the same flavor and their dilepton invariant mass is within 15 GeV of the Z boson mass. If three leptons are found, the two highest-*p*_T_ leptons forming an opposite-sign pair are selected. Jets must satisfy *p*_T_>30 GeV after correcting for additive effects of pileup (multiple proton collisions in a single crossing) and multiplicative effects of jet energy scale calibration. Jets are further required to lie within |*η*|<2.5, where *η* is the pseudorapidity variable, *η*≡−ln[tan(*θ*/2)]. The b-tagging algorithm is the Combined Secondary Vertex (CSV) tagger of Ref. [23], deployed here with an operating point that yields a tagging efficiency of 85 % and mistag rate of 10 %. The mistag rate measures the probability for a light quark or gluon jet to be misidentified as a b jet. In the subsample of events passing all selection requirements of this analysis the b-jet purity is 91 %. Jet masses are required to satisfy a very loose requirement *m*_jet_<40 GeV to assure the existence of kinematic solutions and reject poorly reconstructed jets. The missing transverse energy must satisfy $E_{\mathrm{T}}^{\text{miss}}> 30 ~\text{GeV} $ for e^+^e^−^ and $\mathrm{\mu^{+}} \mathrm{\mu ^{-}} $ events and $E_{\mathrm{T}}^{\text{miss}}>20 ~\text{GeV} $ for $\mathrm{e} ^{\pm} \mathrm{\mu} ^{\mp}$ events, where Drell–Yan backgrounds are smaller. With the exception of the b-tagging criteria and the b-jet mass requirement, all selection requirements summarized here are discussed in more detail in [24, 25]. The sample of events in data meeting all of the signal selection criteria contains 8700 events.

## Kinematic variables

The endpoint method of mass extraction is based on several variables that are designed for use in the kinematically complex environment of events with two cascade decays, each ending in an invisible particle. The challenge here is two-fold, combining the complications of a many-body decay with the limitations of an underconstrained system. In a two-body decay *A*→ *B* *C*, the momentum of either daughter in the parent rest frame exhibits a simple and direct relationship to the parent mass. In a three-body decay, *A*→ *B* *C* *D*, the relationship is less direct, encoded not in a delta function of momentum but in the kinematic boundary of the daughters’ phase space. In general, the parent mass may be determined from the endpoints of the observable daughter momenta in the parent rest frame. To carry out this program, however, the daughter masses must be known and enough of the momenta be measurable or constrained by conservation laws to solve the kinematic equations.

Applying this program to the measurement of the top-quark mass in the decay t→b*ℓν*, one immediately encounters a number of obstacles. At a hadron collider, the $\mathrm{t}\overline{\mathrm {t}}$ system is produced with unknown center-of-mass energy and has an event-dependent *p*_T_-boost due to recoil from the initial-state radiation (ISR). Furthermore, in pp collisions we can apply constraints of momentum conservation only in the two dimensions transverse to the beam direction. Since top quarks are normally produced in pairs, the individual neutrino momenta are indeterminate, adding further complication. These obstacles seem daunting but can be overcome by the use of “designer” kinematic variables *M*_T2_ [12] and *M*_CT_ [26], which, by construction, address precisely these issues. In this paper we use *M*_T2_. Because the transverse momentum of the $\mathrm{t}\overline{\mathrm{t}}$ system varies from event to event, the *p*_T_-insensitive version [27, 28], *M*_T2⊥_, is particularly useful. To measure the masses of the top-quark, W-boson, and neutrino, we measure the endpoints of three kinematic distributions, *μ*_*ℓℓ*_, *μ*_bb_, and *M*_b*ℓ*_, as discussed in the following subsections.

### *M*_T2_ and subsystem variables

#### The *M*_T2_ observable

The variable *M*_T2_ is based on the transverse mass, *M*_T_, which was first introduced to measure the W-boson mass in the decay W→*ℓν*. In this case, *M*_T_ is defined by

4.1 $$ M_{\mathrm{T}} ^{2} \equiv m_{\nu} ^{2} + m_{\ell}^{2} + 2\bigl(E_{\mathrm{T}} ^{\nu}E_{\mathrm{T}} ^{\ell} - \mathbf{p}_{\text{T}} ^{\nu}\cdot \mathbf {p}_{\text{T}} ^{\ell}\bigr). $$

The observable *M*_T_ represents the smallest mass the W boson could have and still give rise to the observed transverse momenta $\mathbf{p}_{\text{T}} ^{\ell}$ and . The utility of *M*_T_ lies in the fact that *M*_T_≤*M*_W_ is guaranteed for W bosons with low transverse momentum. For a single W→*ℓν* decay such a lower limit is only marginally informative, but in an ensemble of events, the maximum value achieved, i.e. the *endpoint* of the *M*_T_ distribution, directly reveals the W boson mass. This observation suggests a “min-max” strategy which is generalized by the invention of *M*_T2_.

The *M*_T2_ observable is useful for finding the minimum parent mass that is consistent with observed kinematics when *two* identical decay chains *a* and *b* each terminate in a missing particle. Figure 1 shows both a SM and a new physics example. If one knew the two missing transverse momenta separately, a value of *M*_T_ could be calculated for either or both of the twin decay chains and the parent mass *M* would satisfy the relationship $\max( M_{\mathrm{T}} ^{\text{a}}, M_{\mathrm{T}} ^{\text{b}}) \le M$. In practice the two missing momenta cannot be known separately, and are observable only in the combination . This compels one to consider all possible partitions of  into two hypothetical constituents $\mathbf{p}_{\text{T}} ^{\text{a}}$ and $\mathbf{p}_{\text{T}} ^{\text{b}}$, evaluating within this ensemble of partitions the *minimum* parent mass *M* consistent with the observed event kinematics. With this extension of the *M*_T_ concept, the variable is now called *M*_T2_: 4.2 As with *M*_T_, the endpoint of the *M*_T2_ distribution has a quantifiable relationship to the parent mass, and the endpoint of an *M*_T2_ distribution is therefore a measure of the unseen parent mass in events with two identical decay chains.

The observable *M*_T2_ requires some care in its use. The presence of $E_{\mathrm{T}} =\sqrt{ p_{\mathrm{T}} ^{2}+m^{2}}$ in Eq. (4.1) implies that one must either know (as in the case of W→*ℓν*) or assume (as in the case of unknown new physics) a value of the mass *m* of the undetected particle(s). In this paper we will refer to an assumed mass as the “test mass” and distinguish it with a tilde (i.e. $\widetilde{m}$); the actual mass of the missing particle, whether known or not, will be referred to as the “true mass”, and written without the tilde. Both the value of *M*_T2_ in any event and the value of the endpoint of the *M*_T2_ distribution in an ensemble of events are in the end *functions* of the test mass.

Even when a test mass has been chosen, however, the endpoint of the *M*_T2_ distribution may not be unique because it is in general sensitive to transverse momentum *P*_T_=|**P**_T_| of the underlying two-parent system, which varies from event to event. The sensitivity vanishes if the test mass can be set equal to the true mass, but such an option will not be immediately available in a study of new physics where the true mass is not known.

The *P*_T_ problem is instead addressed by introducing *M*_T2⊥_ [27, 28], which uses only momentum components transverse to the **P**_T_ boost direction. In this way, *M*_T2⊥_ achieves invariance under **P**_T_ boosts of the underlying two-parent system. The construction of *M*_T2⊥_ is identical to that of *M*_T2_ except that **p**_T_ values that appear explicitly or implicitly in Eq. (4.1) are everywhere replaced by **p**_T⊥_ values, where **p**_T⊥_ is defined to be the component of **p**_T_ in the direction perpendicular to the **P**_T_ of the two-parent system. Formally,

4.3 $$ \mathbf{p}_{\text{T}\perp} \equiv\hat{\mathbf {n}}_{\text{T}}\times ( \mathbf{p}_{\text{T}} \times\hat{ \mathbf {n}}_{\text{T}} ), $$

where $\hat{\mathbf{n}}_{\text{T}}= \mathbf{P}_{\text {T}} /| \mathbf{P}_{\text{T}} |$ is the unit vector parallel to the transverse momentum of the two-parent system.

#### Subsystem variables

A further investigation of *M*_T2_ and *M*_T2⊥_ reveals the full range of kinematic information contained in multistep decay chains by splitting and grouping the elements of the decay chain in independent ways.

The *M*_T2_ variable classifies the particles in an event into three categories: “upstream”, “visible”, and “child”. The child particles are those at the end of the decay chain that are unobservable or simply treated as unobservable; the visible particles are those whose transverse momenta are measured and used in the calculations; and the upstream particles are those from further up the decay chain, including any ISR accompanying the hard collision.

**Fig. 2:**
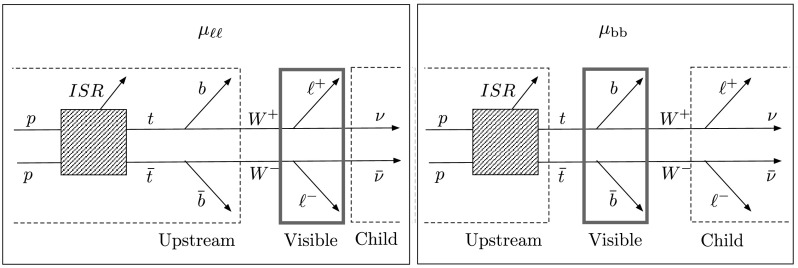
A $\mathrm{t}\overline{\mathrm {t}}$ dilepton decay with the two subsystems for computing *μ*_*ℓℓ*_ and *μ*_bb_ indicated. The “upstream” and “child” objects are enclosed in *dashed rectangles*, while the visible objects, which enter into the computation, are enclosed in *solid rectangles*. The *μ*_*ℓℓ*_ and *μ*_bb_ variables used here are identical to $M_{\mathrm{T}2\perp}^{210} $ and $M_{\mathrm {T}2\perp}^{221} $ of Ref. [10]

In general, the child, visible, and upstream objects may actually be collections of objects, and the subsystem observables introduced in Ref. [10] parcel out the kinematic information in as many independent groupings as possible. Figure 2 shows two of the three possible ways of classifying the $\mathrm{t}\overline{\mathrm{t}}$ daughters for *M*_T2_ calculations. The *μ*_*ℓℓ*_ variable, known as $M_{\mathrm{T}2\perp}^{210}$ in Ref. [10], uses the two leptons of the $\mathrm {t}\overline{\mathrm{t}}$ dilepton decays, treating the neutrinos as lost child particles (which they are), and combining the b jets with all other “upstream” momentum in the event. The *μ*_bb_ variable, known as $M_{\mathrm{T}2\perp}^{221}$ in Ref. [10], uses the b jets, and treats the W bosons as lost child particles (ignoring the fact that their charged daughter leptons are in fact observable). It considers only ISR jets as generators of upstream momentum.

For completeness, we note that a third *M*_T2⊥_ subsystem can be constructed by combining the b jet and the lepton as a single visible system. This variable, known as $M_{\mathrm{T}2\perp }^{220}$ in the nomenclature of Ref. [10], exhibits significant correlation with *M*_b*ℓ*_, the invariant mass of the b jet and lepton. A third observable is needed to solve the underlying system of equations, and for this we choose *M*_b*ℓ*_.

### Observables used in this analysis

This analysis is based on two *M*_T2⊥_ variables, *μ*_*ℓℓ*_ and *μ*_bb_ as described above, and one invariant mass, *M*_b*ℓ*_, the invariant mass of a b jet and lepton from the same top-quark decay. These three quantities have been selected from a larger set of possibilities based on the low correlation we observe among them and the generally favorable shapes of the distributions in their endpoint regions. The observables can be summarized by the underlying kinematics from which they are derived, and the endpoint relations which include the top-quark, W-boson, and neutrino masses.

For the *μ*_*ℓℓ*_ variable, the shape of the distribution is known analytically [27]. In terms of the value *x*=*μ*_*ℓℓ*_ and its kinematic endpoint *x*_max_, the normalized distribution can be written:

4.4 $$ \frac{\mathrm {d}{}N}{\mathrm {d}{}x} = \alpha\delta (x)+(1-\alpha)\frac{4x}{x_{\max}^2}\ln\frac{x_{\max}}{x}, $$

where the parameter *α* is treated as an empirical quantity to be measured. In practice, *α*∼0.6, and the zero bin of *μ*_*ℓℓ*_ histograms will be suppressed to better show the features of the endpoint region. The origin of the delta function is geometric: for massless leptons, *μ*_*ℓℓ*_ vanishes when the two lepton **p**_T⊥_ vectors lie on opposite sides of the axis defined by the upstream **P**_T_ vector, and is equal to $2( p_{\text{T}\perp} ^{\ell^{+}} p_{\text{T}\perp} ^{\ell^{-}})^{1/2}$ otherwise.

For a test mass of the child particle $\widetilde{m}_{\nu}$, the endpoint is related to the masses via [10, 27]:

4.5 $$ \begin{aligned}[b] \mu^{\max}_{{\ell\ell}} & \equiv x_{\max} \\ & = \frac{ M_{ \mathrm{W} } }{2} \biggl(1-\frac{ m^2_{\nu} }{ M^2_{ \mathrm{W} } } \biggr) \\ &\quad {}+ \sqrt{ \frac{ M^2_{ \mathrm{W} } }{4} \biggl(1-\frac{ m^2_{\nu} }{ M^2_{ \mathrm{W} } } \biggr)^2 + \widetilde{m}_\nu ^2}. \end{aligned} $$

In the $\mathrm{t}\overline{\mathrm{t}}$ case, we set the test mass to $\widetilde{m}_{\nu} =0$. We then expect the endpoint at $\mu^{\max}_{{\ell\ell}}= M_{ \mathrm{W} } (1-{ m^{2}_{\nu } }/{ M_{ \mathrm{W} } ^{2}})= M_{ \mathrm{W} } =80.4 ~\text{GeV} $. Note that *m*_*ν*_ is the true mass of the child and *M*_W_ is the true parent mass; these should be viewed as variables in a function for which $\widetilde{m}_{\nu}$ is a parameter. In a new-physics application, the analogs of *M*_W_ and *m*_*ν*_ are not known; but given Eq. (4.5), the measurement of the endpoint, and an arbitrary choice of child mass $\widetilde{m}_{\nu}$, one can fix a *relationship* between the two unknown masses. We emphasize that the equality expressed by Eq. (4.5) holds regardless of the value of the test mass, because the test mass enters into both sides of the equation (see discussion in Sect. 4.1.1). This applies below to Eq. (4.6) also.

In the case of *μ*_bb_, the visible particles are the two b jets, the child particles are the charged leptons and neutrinos (combined), and ISR radiation generates the upstream transverse momentum. We take the visible particle masses to be the observed jet masses, which are typically ∼10 GeV. The endpoint is unaffected by nonzero jet masses provided the test mass is set to the true mass, and is affected only at the ±0.1 GeV level over a large range of test masses, $0< \widetilde {M}_{ \mathrm{W} } < 2 M_{ \mathrm{W} } $. For an assumed child mass $\widetilde{M}_{ \mathrm{W} } $, the endpoint is given by [10, 27]:

4.6 $$ \mu^{\max}_{{\mathrm {b}\mathrm {b}}}=\frac { M_{\mathrm {t}} }{2} \biggl(1-\frac{ M^2_{ \mathrm{W} } }{ M^2_{\mathrm {t}} } \biggr) + \sqrt{\frac{ M^2_{\mathrm {t}} }{4} \biggl(1- \frac { M^2_{ \mathrm{W} } }{ M^2_{\mathrm {t}} } \biggr)^2 + \widetilde{M}_ \mathrm{W} ^2}. $$

In the $\mathrm{t}\overline{\mathrm{t}}$ case, we set the test mass to $\widetilde{M}_{ \mathrm{W} } = M_{ \mathrm{W} } =80.4 ~\text{GeV} $. We then expect the endpoint at $\mu^{\max}_{{\mathrm {b}\mathrm {b}}}= M_{\mathrm {t}} $. As in the previous case, in a new-physics application where the analogs of *M*_t_ and *M*_W_ are not known, the measurement of the endpoint together with an arbitrary choice of the child mass $\widetilde{M}_{ \mathrm{W} } $ yields a relationship between the two unknown masses.

As noted above, a third variable is needed, and we adopt *M*_b*ℓ*_, the invariant mass formed out of jet-lepton pairs emerging from the top-quark decay. Two values of *M*_b*ℓ*_ can be computed in a $\mathrm{t}\overline{\mathrm{t}}$ event, one for each top decay. In practice four are calculated because one does not know a priori how to associate the b jets and leptons; we discuss later an algorithm for mitigating the combinatorial effects on the endpoint. The shape of the distribution is known for correct combinations but is not used here since correct combinations cannot be guaranteed (see Sect. 5.3). The endpoint is given by:

4.7 $$ M^\text{max}_{\mathrm {b}\ell} =\sqrt { m_\mathrm {b}^2 + \biggl( 1-\frac{ m^2_{\nu} }{ M^2_{ \mathrm{W} } } \biggr) \bigl( E^\ast_ \mathrm{W} + p^\ast \bigr) \bigl( E^\ast_\mathrm {b}+ p^\ast \bigr) }, $$

where $E^{\ast}_{ \mathrm{W} }$, $E^{\ast}_{\mathrm {b}}$, and *p* ^∗^ are energies and momenta of the daughters of t→bW in the top-quark rest frame. In these formulae the charged-lepton mass is neglected but the observed b-jet mass *m*_b_ is finite and varies event-to-event.

We can now summarize the mass measurement strategy. If the masses *M*_t_, *M*_W_, and *m*_*ν*_ were unknown, one would measure the two endpoints and the invariant mass that appear on the left-hand sides of Eqs. (4.5)–(4.7), using arbitrary test mass values for the first two, to obtain three independent equations for the three unknown masses. Then, in principle, one solves for the three masses. In practice, the measurements carry uncertainties and an optimum solution must be determined by a fit. In the case when one or more of the masses is known, a constrained fit can improve the determination of the remaining unknown mass(es).

**Fig. 3:**
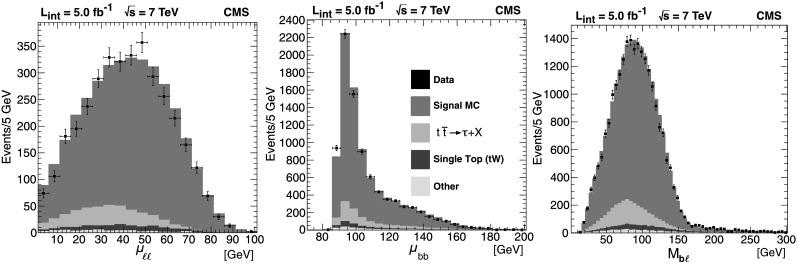
Distributions of the three kinematic distributions *μ*_*ℓℓ*_, *μ*_bb_, and *M*_b*ℓ*_. Data ($5.0\mbox{$~\text{fb}^{\text{$-$1}}$}$) are shown with error bars. MC simulation is overlaid in *solid color* to illustrate the approximate $\mathrm{t}\overline{\mathrm{t}}$ signal and background content of the distributions. The backgrounds contained in “Other” are listed in Table 1. The zero-bin of the *μ*_*ℓℓ*_ plot is suppressed for clarity. The *M*_b*ℓ*_ plot contains multiple entries per event (see Sect. 5.3 for details). In all cases, the simulation is normalized to an integrated luminosity of 5.0 fb^−1^ with next-to-leading-order (NLO) cross sections as described in the text

In Fig. 3 we show distributions for the three observables *μ*_*ℓℓ*_, *μ*_bb_, and *M*_b*ℓ*_. Here and throughout this paper, the zero bin of the *μ*_*ℓℓ*_ distribution, corresponding to the delta function of Eq. (4.4), is suppressed to emphasize the kinematically interesting component of the shape. In the *μ*_bb_ plot shown here, the prominent peak that dominates the figure is an analog of the delta function in *μ*_*ℓℓ*_, its substantial width being due to the variable mass of the jets that enter into the *μ*_bb_ calculation. As with the *μ*_*ℓℓ*_ delta function, the peak arises from events where the axis of the upstream **P**_T_ falls between the two visible-object **p**_T_ vectors. In later plots this *μ*_bb_ peak will be suppressed to better reveal the behavior of the distribution in the endpoint region.

The agreement between data and MC is generally good, but the comparisons are for illustration only and the analysis and results that follow do not depend strongly on the MC simulation or its agreement with observation.

## Backgrounds

The two-lepton requirement at the core of the event selection ensures an exceptionally clean sample. Nevertheless a few types of background must be considered, including top-quark decays with *τ*-lepton daughters, pp→tW events, and sub-percent contributions from other sources.

### Physics backgrounds

The physics backgrounds consist of $\mathrm{t}\overline {\mathrm{t}}$ decays that do not conform to the dilepton topology of interest, as well as non-$\mathrm {t}\overline{\mathrm{t}}$ decays. Table 1 shows the estimation of signal and background events in MC simulation. The MC generators used throughout this study are mc@nlo 3.41 [29] for all $\mathrm{t}\overline{\mathrm{t}}$ samples, pythia 6.4 [30] for the diboson samples, and MadGraph 5.1.1.0 [31] for all others. The simulated data samples are normalized to 7 TeV NLO cross sections and an integrated luminosity of 5.0 fb^−1^.

**Table 1:** Estimate of signal and background composition in MC simulation, normalized to an integrated luminosity of 5.0 fb^−1^ and NLO cross sections as described in the text

Process	Number of events
$\mathrm{t}\overline{\mathrm{t}}$ signal (no *τ*)	7000
$\mathrm{t}\overline{\mathrm{t}}$ signal (*τ*→*ℓν*)	1100
Single top ($\mathrm {t} \mathrm{W} ,\bar{\mathrm {t}} \mathrm{W} $)	270
Drell–Yan	77
Hadronic/Semileptonic $\mathrm{t}\overline{\mathrm{t}} $ with misreconstructed lepton(s)	55
Dibosons (WW, ZZ, WZ)	14
W + jets	9

Events in which a top quark decays through a *τ* lepton (e.g. $\mathrm {t}\to \mathrm {b}\tau^{+}\nu _{\tau}\to \mathrm {b}\ell^{+}\nu_{\ell }\bar{\nu}_{\tau}\nu_{\tau}$), constitute about 13 % of the events surviving all selection requirements. From the point of view of event selection, these events are background. The unobserved momentum carried by the extra neutrinos, however, ensures that these events reconstruct to *M*_T2_ and *M*_b*ℓ*_ values below their true values and hence fall below the endpoint of signal events with direct decays to e or $\mathrm{\mu} $ final states. We therefore include these events among the signal sample. This leaves in principle a small distortion to the kinematic shapes, but the distortion is far from the endpoint and its impact on the mass extraction is negligible.

### Modelling the mistag background

**Fig. 4:**
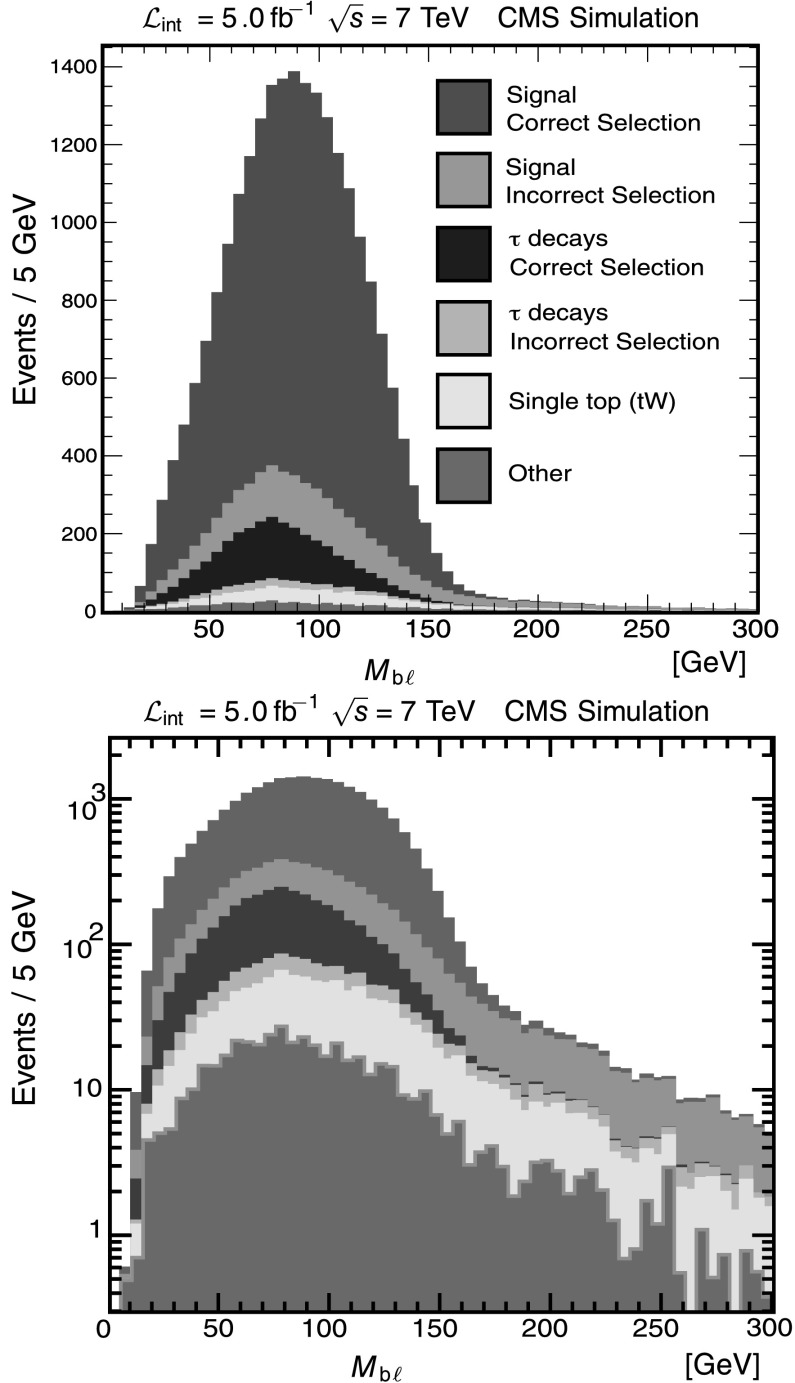
Composition of MC event samples, illustrating that signal events with light-quark and gluon jet contamination dominate the region beyond the endpoint. The *top* and *bottom* *M*_b*ℓ*_ distributions contain the same information plotted with different vertical scales. The *backgrounds* contained in “Other” are listed in Table 1

In addition to the backgrounds discussed above, which fall within the bulk the distributions, it is essential also to treat events that lie beyond the nominal signal endpoint. In this analysis, the main source of such events comes from genuine $\mathrm{t}\overline{\mathrm {t}}$ events where one of the jets not originating from a top-quark decay is mistagged as a b jet. An event in which a light-quark or gluon jet is treated as coming from a top quark can result in events beyond the endpoint in the *μ*_bb_ and *M*_b*ℓ*_ distributions, as can be seen in Fig. 4. The measurement of *μ*_*ℓℓ*_, on the other hand, depends primarily on the two leptons and is unaffected by mistags.

To determine the shape of the mistag background in *μ*_bb_ and *M*_b*ℓ*_, we select a control sample with one b-tagged jet and one antitagged jet, where the antitagging identifies jets that are more likely to be light-quark or gluon jets than b jets. Antitagging uses the same algorithm as combined secondary vertex algorithm, but selects jets with a low discriminator value to obtain a sample dominated by light-quark and gluon jets. We classify event samples by the b-tag values of the two selected jets, and identify three samples of interest: a signal sample where both jets are b-tagged; a background sample where one jet is b-tagged and the other antitagged; and another background sample where both jets are antitagged. Table 2 shows the composition of these samples as determined in MC simulation. We select the sample consisting of pairs with one tagged and one antitagged jet to be the control sample and use it to determine the shape of the background lying beyond the signal endpoint. It contains a significant fraction of signal events, 27 %, but these all lie below the endpoint and categorizing them as background does not change the endpoint fit.

**Table 2:** Composition of b-tagged, dijet samples as determined in MC simulation. Each column is an independently selected sample; columns sum to 100 %

	2 b-tags	b-tag, antitag	2 antitags
b jet, b jet	86 %	27 %	7.1 %
b jet, non b jet	14 %	70 %	53 %
non b jet, non b jet	0.3 %	3 %	40 %

**Fig. 5:**
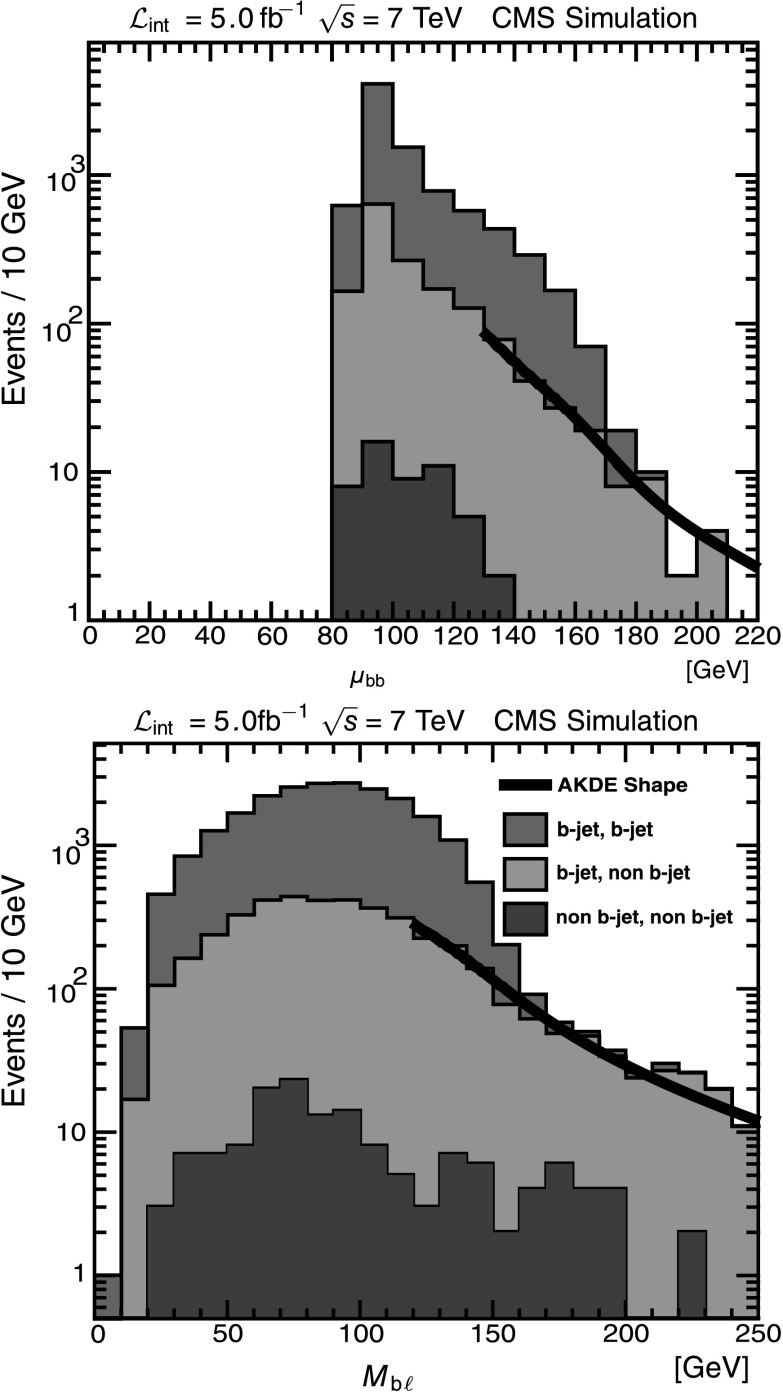
Background PDF shapes determined by the AKDE method, on MC samples. All events pass the signal selection criteria. *Top*: *M*_b*ℓ*_; *bottom*: *μ*_bb_. The *heavy black curve* is the AKDE shape

The control sample is used to generate distributions in *μ*_bb_ and *M*_b*ℓ*_, whose shapes are then characterized with an adaptive kernel density estimation (AKDE) method [32]. The underlying KDE method is a non-parametric shape characterization that uses the actual control sample to estimate the probability distribution function (PDF) for the background by summing event-by-event Gaussian kernels. In the AKDE algorithm, on the other hand, the Gaussian widths depend on the local density of events; empirically this algorithm yields lower bias in the final mass determination than alternative algorithms. Figure 5 shows the performance of the background shape determination; the set of control sample events are taken from MC simulation in order to illustrate the composition of the background and signal.

### Suppressing the combinatorial background

Even if the b-tagging algorithm selected only b jets, there would remain a combinatorics problem in $\mathrm{t}\overline{\mathrm {t}}$ dilepton events. In the case of the *M*_b*ℓ*_ distribution the matching problem arises in pairing the b jet to the lepton: for b jets *j*_1_ and *j*_2_, and leptons *ℓ* ^+^ and *ℓ* ^−^, two pairings are possible: *j*_1_ *ℓ* ^+^,*j*_2_ *ℓ* ^−^ and *j*_1_ *ℓ* ^−^,*j*_2_ *ℓ* ^+^. Four values of *M*_b*ℓ*_ will thus be available in every event, but only two of them are correct. The two incorrect pairings can (but do not have to) generate values of *M*_b*ℓ*_ beyond the kinematic endpoint of *M*_b*ℓ*_ in top-quark decay. To minimize the unwanted background of incorrect pairings while maximizing the chance of retaining the highest values of *M*_b*ℓ*_ in correct b*ℓ* pairings, which do respect the endpoint, we employ the following algorithm.

Let *A* and *a* denote the two *M*_b*ℓ*_ values calculated from one of the two possible b*ℓ* pairings, and let *B* and *b* denote the *M*_b*ℓ*_ values calculated from the other pairing. Choose the labeling such that *a*<*A* and *b*<*B*. Without making any assumptions about which pairing is correct, one can order the *M*_b*ℓ*_ values from smallest to largest; there are six possible orderings. For example the ordering *b*,*B*,*a*,*A* means that the *bB* pairing has *M*_b*ℓ*_ values which are both smaller than the *M*_b*ℓ*_ values in the *aA* pairing. In this case, while we do not know which pairing is *correct*, we can be certain that both *M*_b*ℓ*_ values of the *bB* pairing must respect the true endpoint since either (a) *bB* is a correct pairing, in which case its *M*_b*ℓ*_ values naturally lie below the endpoint, or (b) *aA* is the correct pairing, so its *M*_b*ℓ*_ values lie below the true endpoint, with the *bB* values falling at yet lower values. Similar arguments apply to each of the other possible orderings.

Table 3 shows the six possibilities. For each mass ordering shown in the left column, the right column shows the mass values that will be selected for use in the *M*_b*ℓ*_ fit. For any given event only one row of the table applies. For an event falling in one of the first two rows, two values of *M*_b*ℓ*_ enter in the subsequent fits; for an event falling in the last four rows, three values enter the fits.

**Table 3:** *M*_b*ℓ*_ orderings: in *each column* the left-to-right sequencing of the *a*,*A*,*b*,*B* labels is from lowest *M*_b*ℓ*_ value to highest. The *left column* lists the six possible *M*_b*ℓ*_ orderings; the *right column* indicates for each ordering which values are selected for inclusion in the *M*_b*ℓ*_ plot

Ordering	Selection
*bBaA*	*b*,*B*
*aAbB*	*a*,*A*
*baBA*	*b*,*a*,*B*
*baAB*	*b*,*a*,*A*
*abBA*	*a*,*b*,*B*
*abAB*	*a*,*b*,*A*

This selection algorithm ensures that all masses used in the fits that can be guaranteed to be below the endpoint will be used, while any that *could* exceed the endpoint because of wrong pairings will be ignored. Note that it does not guarantee that the masses that are used are all from correct b*ℓ* pairings; in practice, however, we find that 83 % of the entries in the fit region are correct b*ℓ* pairings, and that this fraction rises to over 90 % within 10 GeV of the endpoint.

## Fit strategy

The kinematic observables *μ*_*ℓℓ*_, *μ*_bb_, and *M*_b*ℓ*_, along with their endpoint relations (Sect. 4.2) and background mitigation techniques (Sects. 5.2, 5.3), are combined in an unbinned event-by-event maximum likelihood fit. The likelihood function is given by a product over all events of individual event likelihoods defined on each of the kinematic variables:

6.1 $$ \mathcal{L}(\mathbf{M})=\prod_{i=1}^{N} { \mathcal{L}}_i^{ \mu_{{\ell\ell}} }(\mathbf {u}_i|\mathbf{M}) \cdot {\mathcal{L}}_i^{ \mu_{{\mathrm {b}\mathrm {b}}} }(\mathbf{u}_i| \mathbf{M})\cdot {\mathcal{L}}_i^{ M_{\mathrm {b}\ell} }(\mathbf {u}_i|\mathbf{M}). $$

The vector $\mathbf{M}=( M_{\mathrm {t}} , M_{ \mathrm{W} } , m^{2}_{\nu } )$ contains the mass parameters to be determined by the fit, and each **u**_*i*_ comprises the set of transverse momentum vectors, reconstructed object masses, and missing-particle test masses from which the kinematic observables *μ*_*ℓℓ*_, *μ*_bb_, and *M*_b*ℓ*_ of the event *i* are computed. We fit for $m^{2}_{\nu}$ rather than *m*_*ν*_ because only $m^{2}_{\nu}$ appears in the endpoint formulae (Eqs. (4.5) and (4.7)); we do not constrain $m^{2}_{\nu}$ to be positive. As will be described more fully below, only the endpoint region of each variable is used in the fit. If an event *i* does not fall within the endpoint region of a given variable, the corresponding likelihood component ($\mathcal{L}_{i}^{ \mu_{{\ell\ell }} }$, $\mathcal {L}_{i}^{ \mu_{{\mathrm {b}\mathrm {b}}} }$, or $\mathcal{L}_{i}^{ M_{\mathrm {b}\ell} }$) defaults to unity.

For each observable *x*∈{*μ*_*ℓℓ*_,*μ*_bb_,*M*_b*ℓ*_}, the likelihood component ${\mathcal{L}}_{i}$ in Eq. (6.1) can be expressed in terms of the value of the observable itself, *x*_*i*_=*x*(**u**_*i*_), and its kinematic endpoint, *x*_max_=*x*_max_(**M**). Explicit formulae for *x*_max_(**M**) are given in Eqs. (4.5), (4.6), and (4.7); in the first two cases there is additional dependence on the missing-particle test mass. Letting the label a∈{*ℓℓ*,bb,b*ℓ*} index the three flavors of observables, we can write the signal, background, and resolution shapes as $S(x|x^{\text{a}}_{\max})$, *B* ^a^(*x*), and $\mathcal{R}^{\text{a}}_{i}(x)$. While the form of the signal shape *S*(*x*) is common to all three fits, the background shape *B* ^a^(*x*) is specific to each observable and the resolution function $\mathcal{R}^{\text{a}}_{i}(x)$ is specific to both the observable and the individual event. Then each function $\mathcal{L}^{\text{a}}_{i}$ appearing on the right-hand side of Eq. (6.1) is given by the general form:

6.2 $$ {\mathcal{L}}^{\text{a}}_i \bigl(x_i|x^\text{a}_{\max}\bigr) = \beta\! \int\! \! S\bigl(y|x^\text{a}_{\max}\bigr) \mathcal{R}^{\text{a}}_i(x_i-y) \, \mathrm {d} {}y + (1-\beta) B^{\text{a}}(x_i). $$

The fit parameter *β* determines the relative contribution of signal and mistag background.

For the common signal shape $S(x|x^{\text{a}}_{\max})$ we use an approximation consisting of a kinked-line shape, constructed piecewise from a descending straight line in the region just below the endpoint and a constant zero value above the endpoint. The kinked-line function is defined over a range from *x*_lo_ to *x*_hi_. The generic form is:

6.3 $$ S(x|x_{\max})\equiv \left \{ \begin{array}{l@{\quad}l} \mathcal{N} (x_{\max} -x) & x_{\text{lo}} \le x \le x_{\max}; \\ 0 & x_{\max}\le x \le x_{\text{hi}}. \end{array} \right . $$

The parameter $\mathcal{N}$ is fixed by normalization. The fidelity of this first-order approximation to the underlying shape depends on both the shape and the value of *x*_lo_. The range of the fit, (*x*_lo_,*x*_hi_), is chosen to minimize the dependence of the fit results on the range, and then the values of *x*_lo_ and *x*_hi_ are subsequently varied to estimate the corresponding systematic uncertainties.

The following paragraphs discuss the forms of *B* ^a^(*x*) and ${\mathcal{R}}^{\text{a}}(x)$ for each of the three kinematic distributions.

### *μ*_*ℓℓ*_

In the case of *μ*_*ℓℓ*_, the visible particles are the two leptons, which are well measured. The projection of their vectors onto the axis orthogonal to the upstream **P**_T_, however, necessarily involves the direction of the upstream **P**_T_, which is not nearly as well determined. The resolution function is therefore wholly dominated by the angular uncertainty in **P**_T_, and it varies substantially from event to event depending on the particular configuration of jets found in each event. Although jet resolutions are known to have small non-Gaussian tails, their impact on the *μ*_*ℓℓ*_ resolution function and the subsequent fit procedure is small and we treat only the Gaussian core. A far more important feature of the resolution arises when the **P**_T_ direction uncertainty is propagated into the *μ*_*ℓℓ*_ variable to derive ${\mathcal{R}}^{{\ell\ell}}_{i}(x)$. In this procedure a sharp Jacobian peak appears wherever the **P**_T_ smearing can cause *μ*_*ℓℓ*_ to pass through a local maximum or minimum value. These peaks depend only on azimuthal angles and occur at any value of *μ*_*ℓℓ*_. The detailed shape of the highly non-Gaussian *μ*_*ℓℓ*_ resolution and its convolution with the underlying signal shape, as specified in Eq. (6.2), are handled by exact formulae derived analytically (see the Appendix). The background in the *μ*_*ℓℓ*_ distribution is vanishingly small, so we set *B* ^*ℓℓ*^(*x*)=0.

### *μ*_bb_

For *μ*_bb_, the visible particles are the b jets, and since the resolution smearing of both the b jets and the upstream jets defining **P**_T_ are large and of comparable magnitudes, the event-by-event resolution is more complicated than in the *μ*_*ℓℓ*_ case. As a result, no analytic calculation is possible and we instead determine the *μ*_bb_ resolution function, $\mathcal{R}^{{\mathrm {b}\mathrm {b}}}_{i}(x)$, numerically in each event, using the known *p*_T_ and *ϕ* resolution functions for the jets. As with the *μ*_*ℓℓ*_ resolutions, Jacobian peaks appear in the *μ*_bb_ resolutions. The mistag background is included by scaling the shape *B* ^bb^(*x*) obtained from the AKDE procedure as discussed in Sect. 5.2.

### *M*_b*ℓ*_

In the *M*_b*ℓ*_ case, the theoretical shape *S*(*x*) is well-known, but the combinatorics of b*ℓ* matching, together with the method of selecting b*ℓ* pairs from the available choices (see Sect. 5.3), sculpt the distribution to the degree that the theoretical shape is no longer useful. Therefore we use the kinked-line shape of Eq. (6.3) to model the signal near the endpoint. In contrast to the *μ*_*ℓℓ*_ and *μ*_bb_ variables, numerical studies confirm that linearly propagated Gaussian resolutions accurately reflect the smearing ${\mathcal{R}}^{{\mathrm {b}\ell}}_{i}(x)$ of *M*_b*ℓ*_, as one expects in this case. The background shape *B* ^b*ℓ*^(*x*) is given by the AKDE procedure as discussed in Sect. 5.2.

### Applying the fit to data

The unbinned likelihood fit prescribed in Eqs. (6.1) and (6.2) is performed on the three kinematic distributions using the shapes given for signal *S*(*x*|*x*_max_), resolution ${\mathcal{R}}_{i}(x)$, and mistag background *B*(*x*). Although a simultaneous fit for all three masses is an important goal of this study, it is useful in the context of the $\mathrm{t}\overline{\mathrm{t}}$ data sample to explore subclasses of the fit in which some masses are constrained to their known values. For this purpose we define: (a) the *unconstrained* fit, in which all three masses are fit simultaneously; (b) the *singly-constrained* fit, in which *m*_*ν*_=0 is imposed; and (c) the *doubly-constrained* fit, in which both *m*_*ν*_=0 and *M*_W_=80.4 GeV are imposed [33]. The unconstrained fit is well-suited to testing mass measurement techniques for new physics, while the doubly-constrained fit is optimal for a SM determination of the top-quark mass.

The fit procedure takes advantage of bootstrapping techniques [34]. In particle physics, bootstrapping is typically encountered in situations involving limited MC samples, but it can be profitably applied to a single data sample, as in this analysis. The goal of bootstrapping is to obtain the sampling distribution of a statistic of a particular data set from the data set itself. With the distribution in hand, related quantities such as the mean and variance of the statistic are readily computable.

In order to estimate the sampling distribution of a statistic, we first need to estimate the distribution from which the data set was drawn. The basic assumption of bootstrapping is that the best estimate for this distribution is given by a normalized sum of delta functions, one for each data point. This is the bootstrap distribution. One can estimate the distribution of a statistic of the data by drawing samples from the bootstrap distribution and calculating the statistic on each sample. To simplify the process further we note that, since the bootstrap distribution is composed of a delta function at each data point, sampling from the bootstrap distribution is equivalent to sampling from the observed data.

**Fig. 6:**
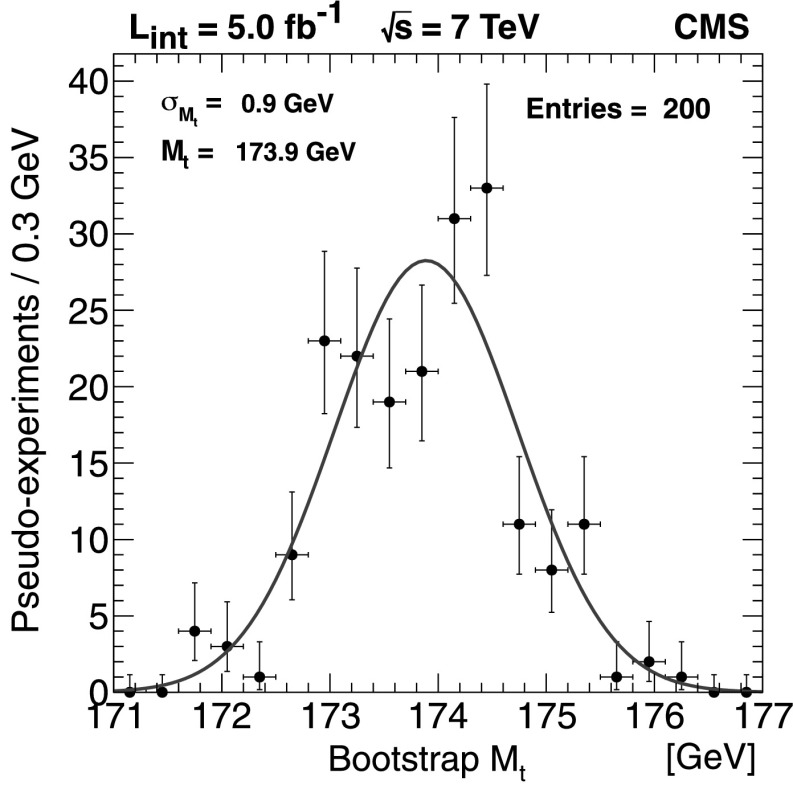
Distribution of *M*_t_ in doubly-constrained fits of 200 pseudo-experiments bootstrapped from the full data set

In this analysis, the fitted top-quark mass is the statistic of interest, and we wish to find its mean and standard deviation. To do so, we conduct the fit 200 times, each time extracting a new sampling of events from the 8700 selected events in the signal region of the full data set. The sampling is done *with replacement*, which means that each of these bootstrapped pseudo-experiments has the same number of events (*N*=8700) as the original data set, and any given event may appear in the bootstrap sample more than once. Each bootstrapped sample is fit with the unbinned likelihood method described in the previous subsections. As an illustration, we show in Fig. 6 the distribution of the 200 values of *M*_t_ that emerges in the case of the doubly-constrained fit; the mean and its standard deviation in this distribution, *M*_t_=173.9±0.9 GeV, constitute the final result of the doubly-constrained fit.

A key motivation for applying bootstrapping to the data is that the impact of possible fluctuations in the background shape are naturally incorporated. Because the background shape in a given fit is constructed from a control sample with the AKDE method (Sect. 5.2), the possible statistical variation in the shape is most easily accounted for by multiple samplings of the control sample. Thus for each bootstrap sample taken from the signal region of the data, another is taken simultaneously from the set of background control events. Each pseudo-experiment fit therefore has its own background function and the ensemble of all 200 such fits automatically includes background shape uncertainties. (The total background yield is a separate issue, handled by the normalization parameter that floats in each fit.)

A secondary motivation to use bootstrapping on the data sample is that it offers a convenient mechanism to correct for event selection and reconstruction efficiencies [35]. To do so, each event is assigned a sampling weight equal to the inverse of its efficiency, and during the bootstrap process events are selected with probabilities proportional to these weights. A bootstrapped data set therefore looks like efficiency-corrected data, but each event is whole and unweighted.

## Validation

We test for bias in the above procedures by performing pseudo-experiments on simulated events. Each pseudo-experiment yields a measurement and its uncertainty for *M*_t_. From these a pull can be calculated, defined by $\text{pull} = ({m_{\text{meas}}-m_{\text{gen}}})/{\sigma_{\text{meas}}}$. In this expression *m*_gen_ is the top-quark mass used in generating events while *m*_meas_ and *σ*_meas_ are the fitted mass and its uncertainty, determined for each pseudo-experiment. For an unbiased fit, the pull distribution will be a Gaussian of unit width and zero mean. A non-zero mean indicates the method is biased, while a non-unit width indicates that the uncertainty is over- or under-estimated. We increase the precision with which we determine the pull distribution width by bootstrapping the simulation to generate multiple pseudo-experiments. The results of Ref. [36] are then used to calculate the mean and standard deviation of the pull distribution, along with uncertainties on each, taking into account the correlations between pseudo-experiments introduced by over-sampling.

**Fig. 7:**
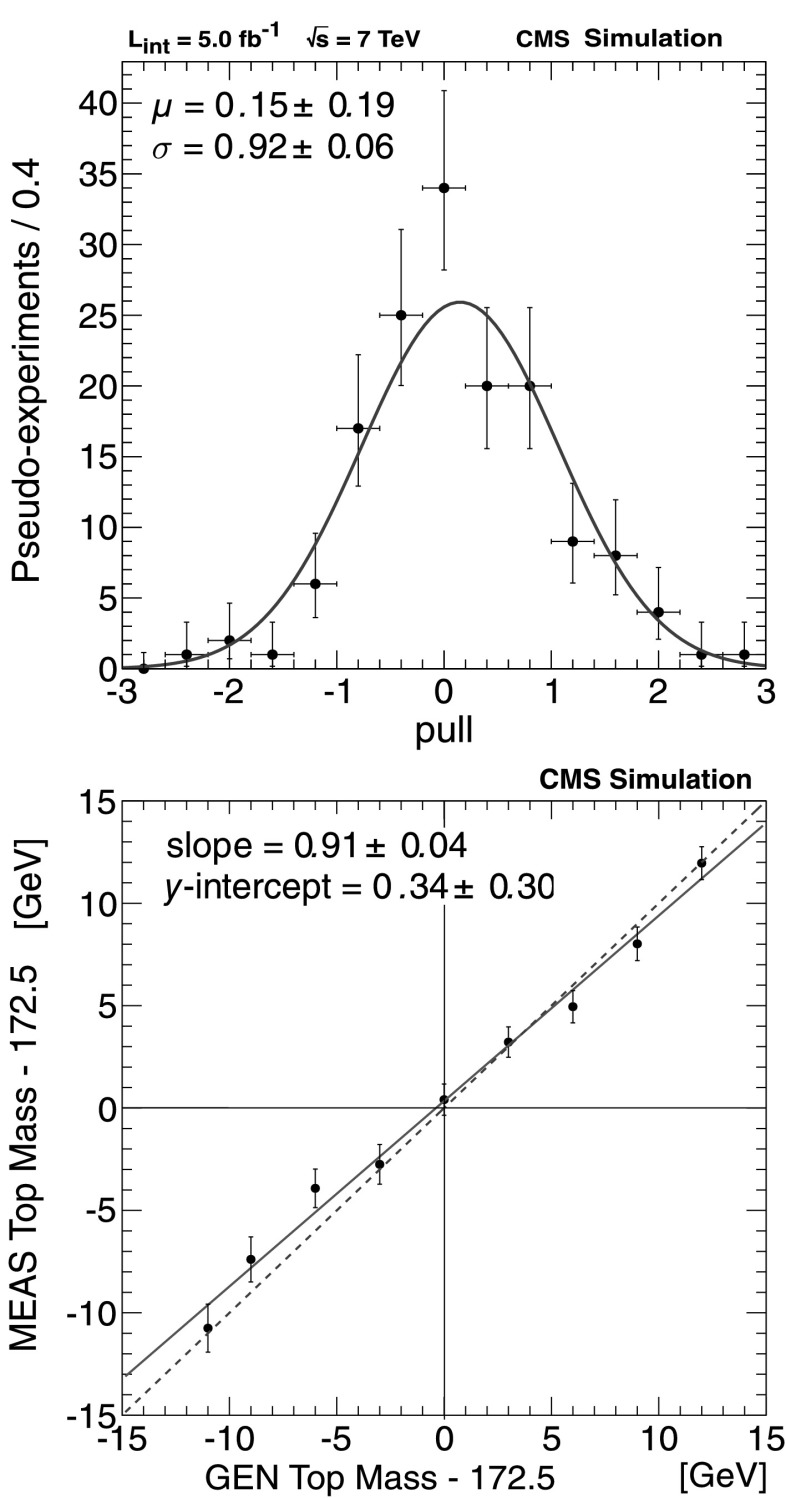
(*Top*) Pull distribution ${(m_{\text{meas}}-m_{\text{gen}})}/{\sigma_{\text{meas}}}$ for the top-quark mass (other masses are fixed) across 150 MC pseudo-experiments. (*Bottom*) Fit results obtained in MC $\mathrm{t}\overline{\mathrm{t}}$-only samples generated with MadGraph for various top-quark masses. The best-fit calibration is shown by the *solid line* and the line of unit slope is shown in the *dashed line*. Data points are from doubly-constrained fits. The line of unit slope agrees with the fit results with *χ* ^2^/degree of freedom=10.7/9

Figure 7, top, shows the pull distribution for the doubly-constrained fit over 150 pseudo-experiments. Extracting a result from each pseudo-experiment involves the methods discussed in Sect. 6.4, and thus the total number of pseudo-experiments required for the study is 150×200. The measured pull mean is 0.15±0.19 and the pull standard deviation is 0.92±0.06, indicating that the fit is unbiased to the level at which it can be measured with the available simulated data. The slightly low standard deviation suggests that the statistical uncertainty may be overestimated; since the systematic uncertainty is significantly larger than the statistical error, we do not make any correction for this.

In an independent test, we perform fits to MC samples generated with various *M*_t_ values. As the results, shown in Fig. 7, bottom, indicate that there is no significant bias as a function of the top-quark mass, we make no correction.

## Systematic uncertainties

The systematic uncertainties are assessed by varying the relevant aspects of the fit and re-evaluating the result. All experimental systematic uncertainties are estimated in data. In the doubly-constrained fit, uncertainties are evaluated for the fitted top-quark mass *M*_t_.

The systematic uncertainties related to absolute jet energy scale (JES) are derived from the calibration outlined in Ref. [22]. We evaluate the effects of JES uncertainties in this analysis by performing the analysis two additional times: once with the jet energies increased by one standard deviation of the JES, and once with them decreased by the same amount. Each jet is varied by its own JES uncertainty, which varies with the *p*_T_ and *η* values of the jet. In a generic sample of multijet events, selecting jets above 30 GeV, the fractional uncertainty in the JES (averaged over *η*) ranges from 2.8 % at the low end to 1 % at high *p*_T_. The uncertainty is narrowed further by using flavor-specific corrections to b jets. A similar process is carried out for varying the jet energy resolutions by its uncertainties. These variations of jet energy scale and jet energy resolution propagate into uncertainties of ${}^{+1.3}_{-1.8} ~\text{GeV} $ and ±0.5 GeV on the measured top-quark mass, respectively. For the electrons, the absolute energy scale is known to 0.5 % in the barrel region and 1.5 % in the endcap region, while for the muons the uncertainty is 0.2 % throughout the sensitive volume. Varying the lepton energy scale accordingly leads to a systematic uncertainty in *M*_t_ of ${}^{+0.3}_{-0.4} ~\text{GeV} $.

The choice of fit range in *μ*_bb_ and *M*_b*ℓ*_ introduces an uncertainty due to slight deviations from linearity in the descending portion of these distributions. Separately varying the upper and lower ends of the *μ*_bb_ and *M*_b*ℓ*_ fit range gives an estimate of ±0.6 GeV for the systematic uncertainty. The uncertainty is mainly driven by dependence on the lower end of the *μ*_bb_ range. A cross-check study based on the methods of Ref. [37] confirms the estimate.

The AKDE shape which is used to model the mistag background in *μ*_bb_ and *M*_b*ℓ*_ is non-parametric and derived from data. For this reason, the AKDE is not subject to biases stemming from assumptions about the underlying background shape or those inherent in MC simulation. However, one could also model the mistag background with a parametric shape, and we use this alternative as a way to estimate the uncertainty due to background modeling. Based on comparisons among the default AKDE background shape and several parametric alternatives, we assign a systematic uncertainty of ±0.5 GeV.

Efficiency can affect the results of this analysis if it varies across the region of the endpoint in one or more of the kinematic plots. The *M*_b*ℓ*_ observable is sensitive to both b-tagging and lepton efficiency variations, whereas *μ*_bb_ is only sensitive to uncertainties due to b-tagging efficiency. By varying the b-tagging and lepton selection efficiencies by ±1*σ*, including their variation with *p*_T_, we estimate that the effect of the efficiency uncertainty contributes at most ${}^{+0.1}_{-0.2} ~\text{GeV} $ uncertainty to the measured top-quark mass.

The dependence on pileup is estimated by conducting studies of fit performance and results with data samples that have been separated into low-, medium-, and high-pileup subsamples of equal population; these correspond to 2–5, 6–8, and ≥9 vertices, respectively. The dependence is found to be negligible. In addition, direct examination of the variables *μ*_bb_ and *M*_b*ℓ*_ reveals that their correlation with the number of primary vertices is small, with correlation coefficients <43 % and <1 %, respectively.

The sensitivity of the result to uncertainties in QCD calculations is evaluated by generating simulated event samples with varied levels of color-reconnection to beam remnants, renormalization and factorization scale, and jet-parton matching scale. The impact of the variations on *M*_t_ is dominated by the color reconnection effects, which are estimated by comparing the results of simulations performed with two different MC tunes [38], Perugia2011 and Perugia2011noCR. Factor-of-two variations of renormalization and factorization scale and the jet-parton matching scale translate to negligible (<0.1 GeV) variations in the top-quark mass. Uncertainties in the parton distribution functions and relative fractions of different production mechanisms do not affect this analysis. The overall systematic error attributed to QCD uncertainties is ±0.6 GeV on the value of *M*_t_. In quadrature with other systematic uncertainties these simulation-dependent estimates add 0.1 GeV to both the upper and lower systematic uncertainties. This additional contribution reflects theoretical uncertainty in the interpretation of the measurement as a top-quark mass, and unlike other systematic uncertainties in the measurement, is essentially dependent on the reliability of the MC modeling.

For the unconstrained and singly-constrained fits, where the objective is primarily to demonstrate a method, rather than to achieve a precise result, we have limited the investigation of systematic uncertainties to just the evaluation of the jet energy scale and fit range variations, which are known from the doubly-constrained case to be the dominant systematic contributions. Because of this, the systematic uncertainties displayed for these fits are slightly lower than they would be with a fuller treatment of all contributions.

The systematic uncertainties discussed in this section are summarized in Table 4.

## Results and discussion

**Fig. 8:**
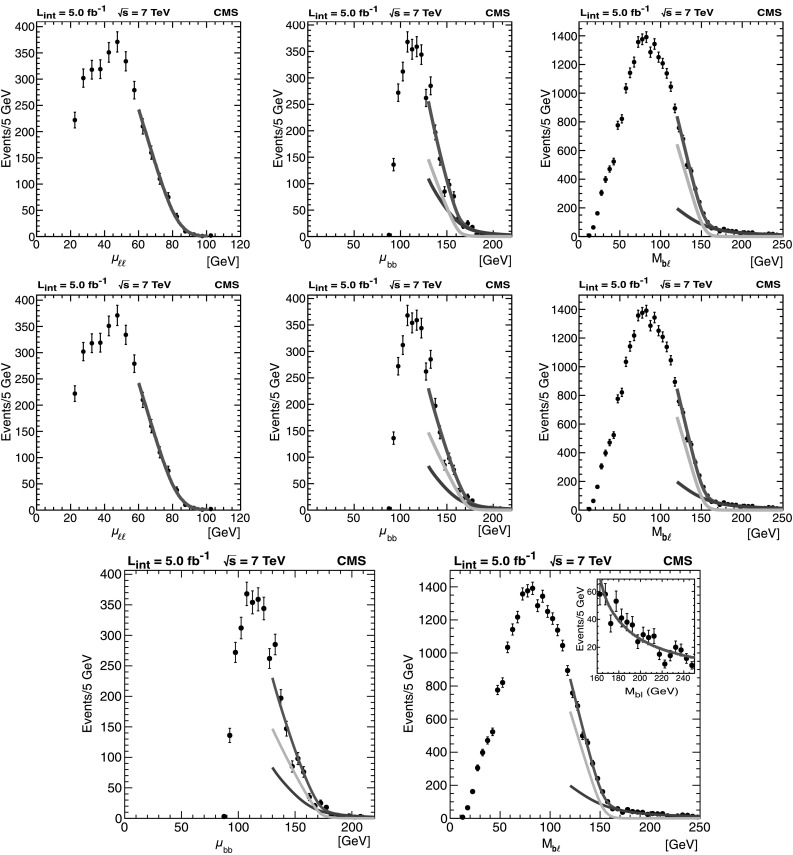
Results of simultaneous fits to $m_{\nu } ^{2}$, *M*_W_, and *M*_t_. The *upper red line* is in all cases the full fit, while the *green* (*middle*) and *blue* (*lowest*) *curves* are for the signal and background shapes, respectively. While the fit is performed event-by-event for all measured kinematic values, the line shown is an approximate extrapolation of the total fit likelihood function over the entire fit range. *Top row*: unconstrained fit; *Middle row*: singly-constrained fit; *Bottom row*: doubly-constrained fit. The *inset* shows a zoom of the tail region in *M*_b*ℓ*_ for the doubly-constrained case to illustrate the level of agreement between the background shape and the data points

The simultaneous fit to the three distributions determines $m^{2}_{\nu}$, *M*_W_, and *M*_t_. A complete summary of central values and statistical and systematic uncertainties for all three mass constraints can be found in Table 5. Figure 8 shows the corresponding fits.

**Table 5:** Fit results from the three mass analyses with various mass constraints. Uncertainties are statistical (*first*) and systematic (*second*). Values in parentheses are constrained in the fit. For the neutrino, squared mass is the natural fit variable—see text for discussion

Fit quantity	Constraint		
	None	*m*_*ν*_=0	*m*_*ν*_=0 and *M*_W_=80.4 GeV
$m^{2}_{\nu} $ (GeV^2^)	−556±473±622	(0)	(0)
*M*_W_ (GeV)	72±7±9	80.7±1.1±0.6	(80.4)
*M*_t_ (GeV)	163±10±11	$174.0\pm 0.9 {}^{+1.7}_{-2.1}$	$173.9\pm0.9 {}^{+1.7}_{-2.1}$

**Table 4:** Summary of systematic uncertainties *δM*_t_ affecting the top-quark mass measurement; see text for discussion

Source	*δM*_t_ (GeV)
Jet energy scale	${}^{+1.3}_{-1.8}$
Jet energy resolution	±0.5
Lepton energy scale	${}^{+0.3}_{-0.4}$
Fit range	±0.6
Background shape	±0.5
Jet and lepton efficiencies	${}^{+0.1}_{-0.2}$
Pileup	<0.1
QCD effects	±0.6
Total	${}^{+1.7}_{-2.1}$

We take the doubly-constrained version to be the final result:

9.1 $$ M_{\mathrm {t}} = 173.9 \pm0.9 ~\text {(stat.)} {}^{+1.7}_{-2.1} ~\text{(syst.)} ~\text{GeV} . $$

In the more general case of the unconstrained measurement, the performance of the endpoint method illustrated here in the $\mathrm {t}\overline{\mathrm{t}}$ dilepton system suggests the technique will be a viable option for mass measurements in a variety of new-physics scenarios. The precision on *M*_t_ given by the doubly-constrained fit, for example, is indicative of the precision with which we might determine the masses of new colored particles (like squarks), *as a function* of the input test mass $\widetilde{m}_{\nu}$. Of course, as shown in the second column of Table 5, the input mass *m*_*ν*_ itself will be determined less precisely. Another plausible scenario is one in which new physics mimics the leptonic decay of the W boson. This can arise in SUSY with *R*-parity violation and a lepton-number violating term in the superpotential. In this case, the lightest superpartner could be the charged slepton, which decays to a lepton and neutrino, just like the SM W boson. Current bounds from LEP indicate that the slepton must be heavier than 100 GeV. Given the ∼1 GeV precision provided by the singly-constrained fit on the W boson mass, the W boson can easily be discriminated from such an object based on its mass.

It is interesting to note also that in the unconstrained case, one can restrict the range of the neutrino mass (which is treated as an unknown parameter) reasonably well, within approximately 20 GeV, in line with previous expectations [39]. If the $E_{\mathrm {T}}^{\text{miss}}$ signal is due to SM neutrinos, rather than heavy WIMPs with masses of order 100 GeV, this level of precision is sufficient to distinguish the two cases. If, on the other hand, the $E_{\mathrm{T}}^{\text{miss}}$ signal is indeed due to heavy WIMPs, one might expect that the precision on the WIMP mass determination will be no worse than what is shown here for the neutrino, assuming comparable levels of signal and background.

## Conclusions

A new technique of mass extraction has been applied to $\mathrm{t}\overline{\mathrm{t}}$ dilepton events. Motivated primarily by future application to new-physics scenarios, the technique is based on endpoint measurements of new kinematic variables. The three mass parameters $m^{2}_{\nu}$, *M*_W_, and *M*_t_ are obtained in a simultaneous fit to three endpoints. In an unconstrained fit to the three masses, the measurement confirms the utility of the techniques proposed for new-physics mass measurements. When $m^{2}_{\nu}$ and *M*_W_ are constrained to 0 and 80.4 GeV respectively, we find $M_{\mathrm {t}} =173.9 \pm0.9 ~\text{(stat.)} {}^{+1.7}_{-2.1} ~\text{(syst.)} ~\text{GeV} $, comparable to other dilepton measurements. This is the first measurement of the top-quark mass with an endpoint method. In addition to providing a novel approach to a traditional problem, it achieves a precision similar to that found in standard methods, and its use lays a foundation for application of similar methods to future studies of new physics.

## Figures and Tables

**Fig. 9:**
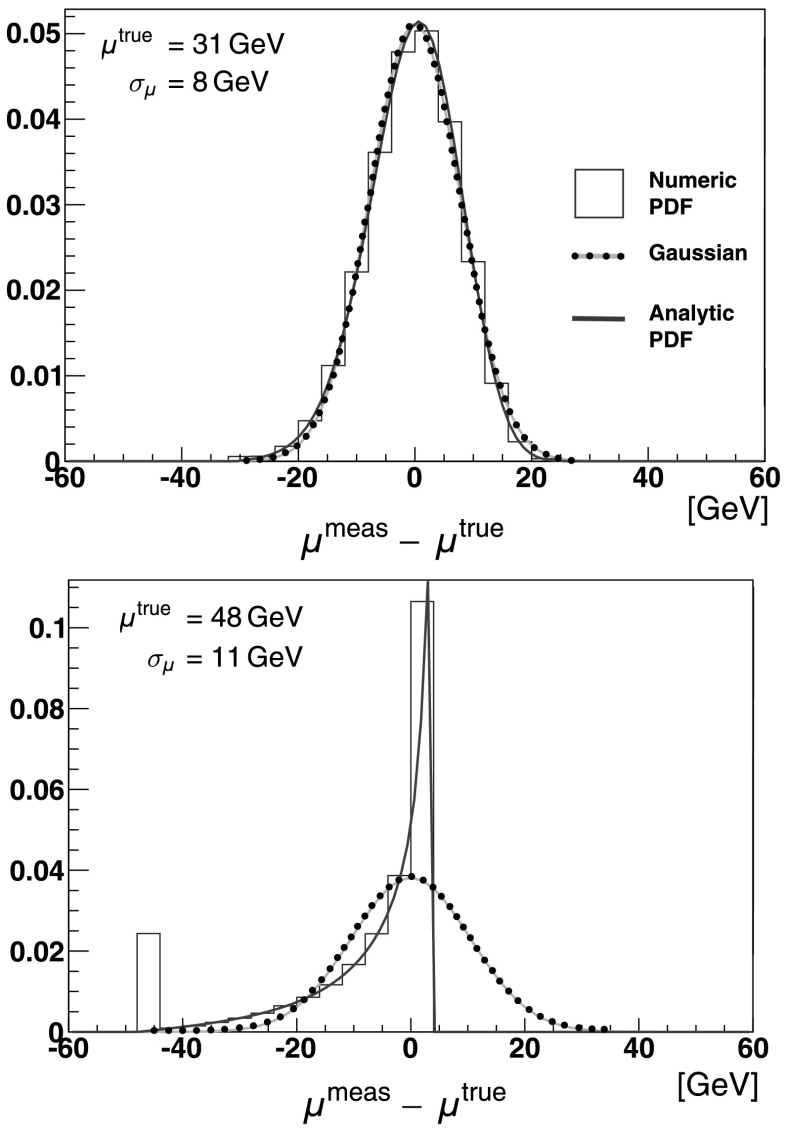
Example resolution functions. The *panels* show two events with different lepton and upstream momentum kinematics, as discussed in the text. The *dotted curve* is a Gaussian with a *σ* given by the linearly propagated uncertainties; and the *solid curve* is the analytic form of the resolution function, given in Eq. (A.4). The histogram is created by numerically propagating resolutions in the underlying parameters

## Appendix: Analytical resolution functions for *μ*_*ℓℓ*_

We present the analytical forms of the resolution functions used in the *μ*_*ℓℓ*_ fits, together with a brief summary of their derivation.

The leptons used in computing *μ*_*ℓℓ*_ are approximately massless and therefore the *M*_*T*2⊥_ variable may be written [27] as

A.1 $$ \begin{aligned}[b] { \mu_{{\ell\ell}} ^2}&= 2(E_{\text{T1}\perp} E_{\text{T2}\perp} -\mathbf{p}_{\text{T1}\perp} \cdot \mathbf{p}_{\text{T2}\perp}) \\ &={2p_{\text{T1}}p_{\text{T2}}} \bigl[ \bigl|\sin(\phi_1-{ \phi_{\text{US}}})\sin(\phi_2-{\phi_{\text{US}}})\bigr| \\ &\quad {}- \sin(\phi_1-{\phi_{\text{US}}})\sin(\phi_2-{ \phi_{\text{US}}}) \bigr], \end{aligned} $$

where (*p*_*Ti*_,*ϕ*_*i*_) are the transverse coordinates of lepton *i*∈{1,2} and ${\phi_{\text{US}}}$ is the azimuthal angle of the upstream momentum in the CMS reference frame.

If the upstream **P**_T_ vector happens to lie *between* the two lepton vectors **p**_T1_ and **p**_T2_, so that $\phi_{1}-{\phi_{\text{US}}}>0$ and $\phi _{2}-{\phi_{\text{US}}}<0$ (or vice-versa) then the value of *μ*_*ℓℓ*_ is identically zero. This is the origin of the delta function in Eq. (4.4). It is convenient to measure ${\phi_{\text{US}}}$ from the midline between the lepton **p**_T_ vectors rather than from the CMS-defined *x* axis, and hence we define ${\varPhi}\equiv {\phi_{\text{US}}}-\frac{1}{2}(\phi_{1}+\phi_{2})$. We also define the separation between the two lepton vectors: Δ*ϕ*≡*ϕ*_1_−*ϕ*_2_.

Equation (A.1) can now be rewritten as:

A.2 $$ \mu({\varPhi})= \begin{cases} \sqrt{{4p_{\text{T}1}p_{\text{T}2}}\sin({\varPhi}-\frac{1}{2}\Delta \phi)\sin({\varPhi}+\frac{1}{2}\Delta\phi)}\\ \hphantom{0}\quad\text{when }|{\varPhi}|>|\frac{1}{2}\Delta\phi|; \\ 0 \quad\text{otherwise}. \end{cases} $$

To streamline the notation, we have dropped the subscript *ℓℓ*. (In any case these remarks apply only to calculations on the *ℓℓ* system.)

The leptonic observables *p*_T1_, *p*_T2_, *ϕ*_1_, and *ϕ*_2_ are well-measured compared to the direction of the upstream jets, *Φ*, and thus the resolution of *μ*(*Φ*) in a given event depends only on the *Φ* resolution, with the leptonic observables treated as fixed parameters. The distribution of *Φ* is well-approximated by a Gaussian form, with *σ*_*Φ*_≪*π*; we ignore small non-Gaussian tails.

The functional form given in Eq. (A.2) is maximal at *Φ*=*π*/2, falls to zero on either side at ${\varPhi}={\pm}\frac{1}{2}\Delta\phi$, and is exactly zero in the neighboring regions $[0,\frac{\pi}{2}-\frac {1}{2}\Delta\phi]$ and $[\frac{\pi}{2}+\frac{1}{2}\Delta\phi,\pi]$. The function is periodic in *Φ* with period *π*, but because of the condition *σ*_*Φ*_≪*π* we restrict our attention to the interval 0≤*Φ*≤*π*. For the non-zero portion of *μ*(*Φ*) there is also an inverse function:

A.3 $$ {\varPhi}(\mu)=\frac{1}{2} \cos^{-1} \biggl[ \frac{\mu^{2}_{\max}-\mu^{2}}{2p_{\text{T1}}p_{\text{T2}}} -1 \biggr]. $$

The inverse function *Φ*(*μ*) is double-valued as one value of *μ* maps to two values of *Φ* located symmetrically on either side of *π*/2. The maximum value of *μ*, here denoted *μ*_max_, is the largest value *μ* can take on for the given the lepton momentum vectors; it corresponds to *Φ*=*π*/2 where the lepton bisector is orthogonal to the upstream momentum. It should not be confused with the endpoint of the *μ* distribution, which, in addition to the upstream momentum orientation *Φ*=*π*/2, also requires extreme lepton kinematics: *p*_T1_ *p*_T2_ maximal and Δ*ϕ*=0 (leptons collinear).

To map the Gaussian PDF *G*(*Φ*|*σ*_*Φ*_) into a resolution function *R*_1_(*μ*), we write:

A.4 $$ {R}_{1}(\mu) = \sum{G\bigl({\varPhi}(\mu)\big| \sigma_{{\varPhi}}\bigr)}\biggl \vert \frac {\mathrm {d}{\varPhi}(\mu)}{\mathrm {d}\mu}\biggr \vert , $$

where the sum is over the two branches of the double-valued *Φ*(*μ*). The derivatives of *Φ*(*μ*) and *μ*(*Φ*) have simple analytic forms.

In the region where *μ*(*Φ*)=0, *R*(*μ*) is a delta function *R*_0_ *δ*(*μ*) whose amplitude *R*_0_ is given by the area under *G*(*Φ*|*σ*_*Φ*_) in the angular region between the two leptons, $R_{0}\equiv\int_{-\Delta\phi/2}^{\Delta\phi/2}G({\varPhi}|\sigma _{{\varPhi}})\,\mathrm {d}{\varPhi}$. Thus the total resolution function is given by

A.5 $$ {R}(\mu) = R_{0}\delta(\mu) + \varTheta(\mu) \sum{G\bigl({ \varPhi}(\mu)\big|\sigma_{{\varPhi}}\bigr)}\biggl \vert \frac{\mathrm {d}{\varPhi}(\mu )}{\mathrm {d}\mu}\biggr \vert , $$

where *Θ*(*μ*) is the unit step function transitioning from 0 to 1 at *μ*=0.

Figure 9 shows two representative cases, showing the range of resolution function behavior from Gaussian to sharply peaked. In the latter case the delta function *R*_0_ *δ*(*μ*) is not plotted. In the top panel the *Φ* is midway between the extremes ${\pm}\frac{1}{2}\Delta\phi$ and *π*/2 and the *σ*_*Φ*_ is relatively narrow; in the bottom panel, *Φ* is closer to *π*/2 and has a large value of *σ*_*Φ*_ that allows smearing into the $-\frac{1}{2}\Delta\phi<{\varPhi}<\frac {1}{2}\Delta\phi$ region. The high bin at −45 GeV in the histogram component of the bottom panel contains events in which the resolution smearing of the upstream momentum vector pushed the *μ*_*ℓℓ*_ value into the delta function at *μ*_*ℓℓ*_=0. In the analytic form, the corresponding feature would be the delta function *R*_0_ *δ*(*μ*); but, as noted above, this has not been explicitly drawn.
